# Deletion of Nkx2-5 in trabecular myocardium reveals the developmental origins of pathological heterogeneity associated with ventricular non-compaction cardiomyopathy

**DOI:** 10.1371/journal.pgen.1007502

**Published:** 2018-07-06

**Authors:** Caroline Choquet, Thi Hong Minh Nguyen, Pierre Sicard, Emeline Buttigieg, Thi Thom Tran, Frank Kober, Isabelle Varlet, Rachel Sturny, Mauro W. Costa, Richard P. Harvey, Catherine Nguyen, Pascal Rihet, Sylvain Richard, Monique Bernard, Robert G. Kelly, Nathalie Lalevée, Lucile Miquerol

**Affiliations:** 1 Aix-Marseille Université, CNRS UMR 7288, IBDM, Marseille, France; 2 Aix-Marseille Université, INSERM UMR 1090, TAGC, Marseille, France; 3 INSERM, CNRS, Université de Montpellier, PHYMEDEXP, Montpellier, France; 4 Aix-Marseille Université, CNRS, CRMBM, Marseille, France; 5 Australian Regenerative Medicine Institute, Monash University, Clayton, Australia; 6 The Jackson Laboratory, Bar Harbor, Maine, United States of America; 7 Developmental and Stem Cell Biology Division, Victor Chang Cardiac Research Institute, Darlinghurst NSW, Australia; 8 St. Vincent’s Clinical School and School of Biological and Biomolecular Sciences, University of New South Wales, Kensington, Australia; Indiana University Purdue University at Indianapolis, UNITED STATES

## Abstract

Left ventricular non-compaction (LVNC) is a rare cardiomyopathy associated with a hypertrabeculated phenotype and a large spectrum of symptoms. It is still unclear whether LVNC results from a defect of ventricular trabeculae development and the mechanistic basis that underlies the varying severity of this pathology is unknown. To investigate these issues, we inactivated the cardiac transcription factor *Nkx2-5* in trabecular myocardium at different stages of trabecular morphogenesis using an inducible *Cx40-creERT2* allele. Conditional deletion of *Nkx2-5* at embryonic stages, during trabecular formation, provokes a severe hypertrabeculated phenotype associated with subendocardial fibrosis and Purkinje fiber hypoplasia. A milder phenotype was observed after *Nkx2-5* deletion at fetal stages, during trabecular compaction. A longitudinal study of cardiac function in adult *Nkx2-5* conditional mutant mice demonstrates that excessive trabeculation is associated with complex ventricular conduction defects, progressively leading to strain defects, and, in 50% of mutant mice, to heart failure. Progressive impaired cardiac function correlates with conduction and strain defects independently of the degree of hypertrabeculation. Transcriptomic analysis of molecular pathways reflects myocardial remodeling with a larger number of differentially expressed genes in the severe *versus* mild phenotype and identifies Six1 as being upregulated in hypertrabeculated hearts. Our results provide insights into the etiology of LVNC and link its pathogenicity with compromised trabecular development including compaction defects and ventricular conduction system hypoplasia.

## Author summary

During fetal heart morphogenesis, formation of the mature ventricular wall requires coordinated compaction of the inner trabecular layer and growth of the outer layer of myocardium. Arrested trabecular development has been implicated in the pathogenesis of hypertrabeculation associated with ventricular non-compaction cardiomyopathy. However much uncertainty still exists among clinicians concerning the physiopathology of ventricular non-compaction cardiomyopathy, including its clinical characteristics, prognosis, classification and even the definition of hypertrabeculation. In particular, distinguishing between pathological and non-pathological subtypes of non-compaction is currently a major issue. Here we show that deletion of the gene encoding the transcription factor Nkx2-5 at critical steps during trabecular development recapitulates pathological features of hypertrabeculation, providing the first model of ventricular non-compaction cardiomyopathy in adult mice. We demonstrate that excessive trabeculation due to failure of trabecular compaction during fetal development is associated with Purkinje fiber hypoplasia and subendocardial fibrosis. Longitudinal functional studies reveal that these mice present all the clinical signs of symptomatic left ventricular non-compaction cardiomyopathy, including conduction defects, strain defects and progressive heart failure. Our results, including transcriptomic analysis, suggest that pathological features of non-compaction are primarily developmental defects. This study clarifies the origin of the pathological outcomes associated with LVNC and may provide helpful information for clinicians concerning the etiology of this rare cardiomyopathy.

## Introduction

Left ventricular non-compaction (LVNC) or hypertrabeculation is a cardiac anomaly characterized by a thickened sub-endocardial layer of ventricular myocardium with prominent trabeculations and deep recesses that communicate with the left ventricular (LV) cavity [1]. The recent classification of LVNC as the third most common form of cardiomyopathy is still debated because of the strong variability in pathophysiology, clinical symptoms and genetic associations [2, 3]. LVNC has been described as a genetic disorder caused by mutations in genes encoding sarcomere, cytoskeletal, nuclear membrane, ionic channels and chaperone proteins [4, 5]. Most importantly, LVNC can be asymptomatic but complications including heart failure (HF), thromboembolism and malignant arrhythmia are often observed [6]. The relatively recent interest of clinicians in LVNC comes from the increasing number of patients presenting signs of hypertrabeculation in the last 25 years, due to significant improvements in cardiac imaging using echocardiography or Magnetic Resonance Imaging (MRI). However, the phenotypic variability of this anomaly raises major problems for clinicians concerning its pathological nature. In particular, the wide spectrum of clinical signs makes prognosis of LVNC difficult. To understand the etiology of this non-compaction the generation of adult mouse models is essential.

The anatomic resemblance between human cases of LVNC and embryonic hearts have suggested that this anomaly results from an arrest of myocardial compaction during fetal development [7]. This hypothesis is now well established in several mouse models [8–11]; however, it does not explain the occurrence of different LVNC subtypes. The presence of ventricular trabeculae is assumed to be critical for oxygenation and conduction in the developing heart [12]. Trabeculation is initiated by the formation of endocardial outpockets in which trabecular myocardium develops by proliferation of cardiomyocytes forming a two-layer myocardium with a compact and a trabecular zone. In contrast to fishes and reptiles, mammalian trabeculae are not maintained in the adult heart, their reduction occurs by a process of compaction that initiates during fetal stages and ends after birth [13]. This is thought to arise by coalescence of trabeculae, which are progressively incorporated to the compact myocardium as well as forming papillary muscles [14]; however, the mechanisms underlying compaction of the ventricular trabeculae remain unknown. Moreover, trabecular development is directly coupled with the development of the ventricular conduction system. The function of the conduction system is to generate and orchestrate the propagation of the electrical activity through the heart in order to coordinate the sequential contraction of atria and ventricles [15]. The gap junction Connexin40 (Cx40) characterized by high conductance properties is strongly expressed in ventricular trabeculae and Cx40+ cardiomyocytes form preferential conduction pathways in the embryonic heart [16].

The cardiac transcription factor Nkx2-5 is an important regulator of ventricular trabeculae and conduction system development. *Nkx2-5*^*+/-*^ haploinsufficient mice have an abnormal electrocardiogram, with a prolonged QRS and progressive elongation of the PR interval [17, 18]. These mice display ventricular conduction defects that can be correlated with hypoplastic development of the His-Purkinje system [19]. More recently, *NKX2-5* mutations have been identified in LVNC patients indicating a role of this transcription factor in compaction [8, 20]. Indeed, conditional inactivation of *Nkx2-5* in the ventricular myocardium showed that this deficiency provokes a hypertrabeculated phenotype resulting from defective myocardial proliferation [10]. In order to investigate the impact of the loss of *Nkx2-5* during trabecular development, we have now conditionally inactivated this gene in the ventricular trabeculae in a spatiotemporally controlled manner using an inducible *Cx40-creERT2* mouse model [21]. This study demonstrates that deletion of *Nkx2-5* during trabecular development recapitulates in adult mice pathological features associated with LVNC and represents a highly tractable model to study the cellular and molecular mechanisms involved in the disease as well as the pathological evolution of LVNC.

## Results

### Spatiotemporal deletion of *Nkx2-5* in the ventricular trabecular myocardium

**Fig 1:**
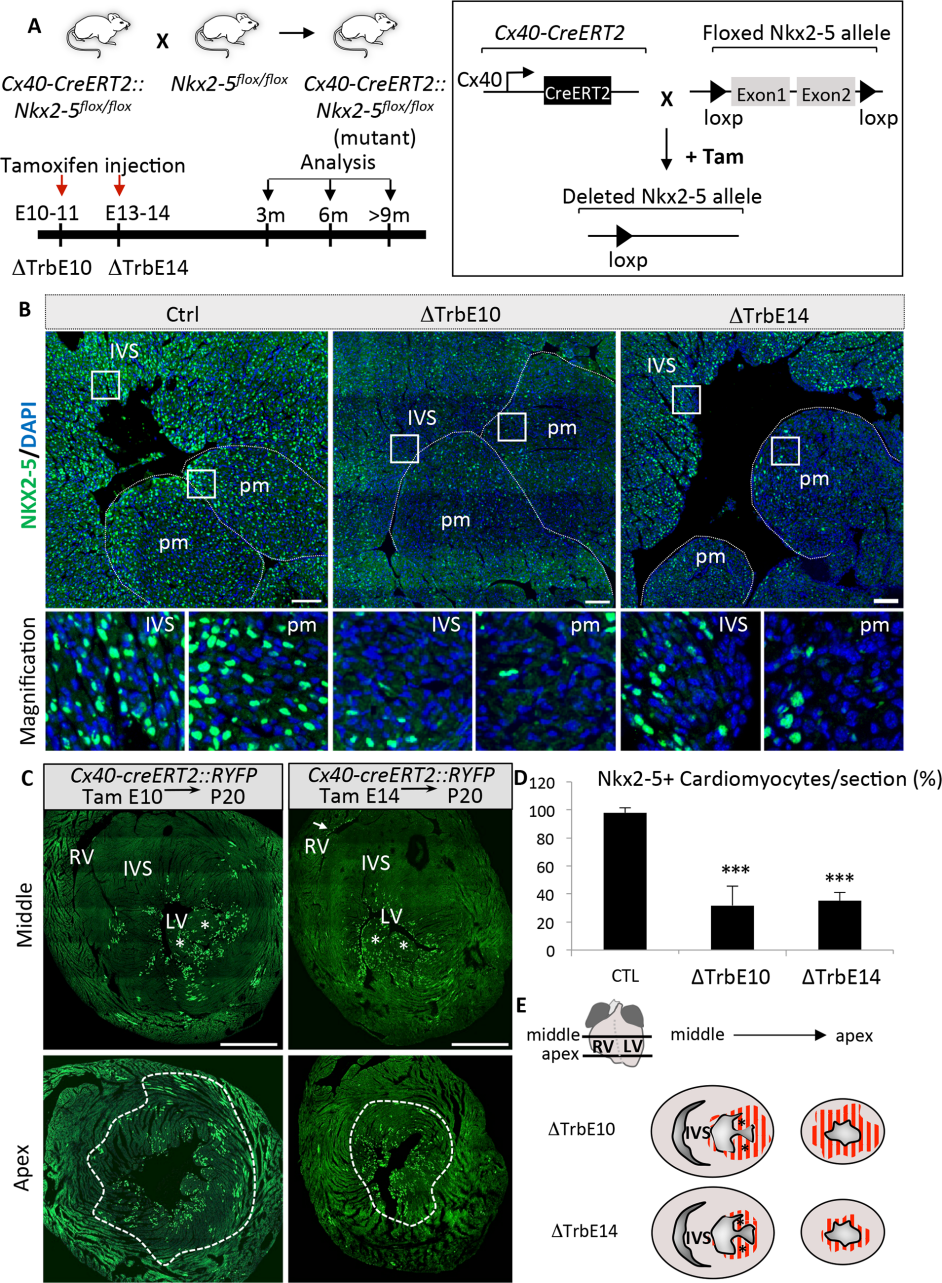
Gene targeting strategy and validation of conditional deletion of *Nkx2-5* in ventricular trabeculae. (A) Schematic illustration of the gene targeting strategy. Conditional *Nkx2-5* inactivation (*Nkx2-5*^*-/-*^*)* was obtained by crossing mice containing an *Nkx2-5* floxed allele with tamoxifen-inducible *Cx40-cre-ERT2* mice. Time course of tamoxifen injections and cardiac phenotyping analyses are represented on a time scale and the corresponding *Nkx2-5* mutant groups, *i*.*e*. ΔTrbE10 and ΔTrbE14 are indicated below the scale. (B) Nkx2-5 immunofluorescence on transversal sections of adult hearts from Control (Ctrl) and *Nkx2-5*^*ΔTrbE10*^ (∆TrbE10) and *Nkx2-5*^*ΔTrbE14*^ (∆TrbE14) mutant mice. (C) Genetic tracing of ventricular trabeculae in *Cx40-creERT2*::*R26-YFP* mice at P20 showing the distribution of YFP+ in the apex and mid ventricle after Cre induction at E10 or E14. (D) *Nkx2-5* deletion was quantified by counting the percentage of Nkx2-5-positive cardiomyocytes per frame at the subendocardial surface of transverse sections of adult Control (CTL), *Nkx2-5*^*ΔTrbE10*^ (∆TrbE10) and *Nkx2-5*^*ΔTrbE14*^ (∆TrbE14) mutant mice. n = 20–30 frames per heart; N = 3 mice per group; Mean±SEM; ***p<0.001 *Nkx2-5*^*ΔTrbE10*^ *vs* control, ***p<0.001 *Nkx2-5*^*ΔTrbE14*^ *vs* control (E) Schematic representation of the *Nkx2-5*-deleted cardiac regions in the apex or in mid-ventricle after tamoxifen injection at embryonic stages (∆TrbE10) or fetal stages (∆TrbE14). IVS: interventricular septum; pm: papillary muscle.

Our strategy to conditionally inactivate *Nkx2-5* in the developing ventricular trabeculae is summarized in Fig 1A. We crossed mice containing *Floxed-Nkx2-5-Δneo* alleles with *Cx40-creERT2* mice in which Cre recombinase is broadly expressed in embryonic trabecular myocardium [21, 22]. Tamoxifen was injected into pregnant females at embryonic (E10.5-E11.5) or fetal (E13.5-E14.5) stages to remove *Nkx2-5* during the formation of ventricular trabeculae (*Nkx2-5*^*ΔTrbE10*^) or during the step of trabecular compaction (*Nkx2-5*^*ΔTrbE14*^) respectively.

The efficiency of *Nkx2-5* deletion was verified by immunofluorescence on sections from control, *Nkx2-5*^*ΔTrbE10*^ and *Nkx2-5*^*ΔTrbE14*^ mutant hearts. Numerous Nkx2-5-negative cardiomyocytes were observed in the apex, the papillary muscles and left ventricular free wall after tamoxifen injection at embryonic or fetal stages (Fig 1B). We performed a genetic tracing analysis of *Cx40-cre* derived cells to evaluate the extent of cardiac tissue affected by *Nkx2-5* deletion. We crossed *Cx40-cre* mice with *Rosa26-YFP* reporter mice and observed the localization of YFP-positive cells in adult hearts by immunofluorescence on sections after tamoxifen induction at E10 or E14 (Fig 1C). YFP-positive cardiomyocytes were observed throughout the left ventricle, including compact zone myocardium, after induction at E10 while YFP+ cells are restricted to the inner half of the LV wall after induction at fetal stages, consistent with previous observations [16]. Almost no YFP staining was detected in the RV, in accordance with the low level of expression of Cx40 in this ventricle. These data are consistent with a contribution of ventricular trabeculae at embryonic and fetal stages to the morphogenesis of the papillary muscles and ventricular free wall with limited contributions to the interventricular septum. Quantification of Nkx2-5-positive subendocardial cardiomyocytes shows a significant reduction in *Nkx2-5*^*ΔTrbE10*^ and *Nkx2-5*^*ΔTrbE14*^ mutants after two consecutive tamoxifen injections at embryonic or fetal stages (Fig 1D). Consistent with the dynamic expression profile of *Cx40* and our genetic tracing results, Nkx2-5 is deleted in a large proportion of the ventricular myocardium when tamoxifen was injected at embryonic stages, while deletion is restricted to the subendocardial zone at fetal stages (Fig 1E). These data suggest that the participation of *Cx40*-derived trabecular cells to the ventricular wall is reduced as trabecular compaction progresses during cardiac development.

### Temporal deletion of *Nkx2-5* induces hypertrabeculation accompanied by deep endocardial recesses

**Fig 2:**
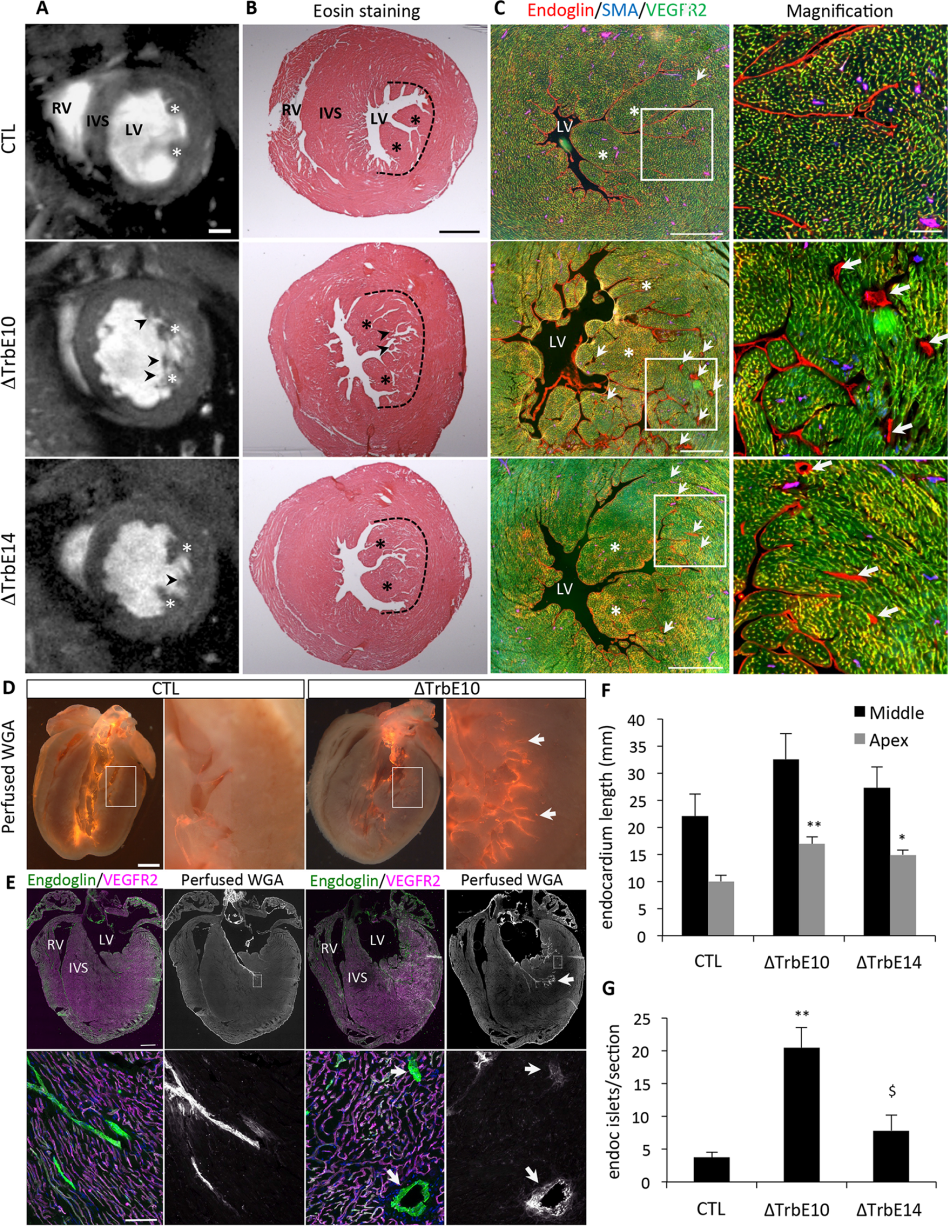
Morphology of adult hypertrabeculation induced by conditional deletion of *Nkx2-5* in cardiomyocytes during trabecular development. (A) Short-axis cine images recorded by CMR at end-diastole show anatomical structures of LV of 3 months-old control (CTL), *Nkx2-5*^*ΔTrbE10*^ (ΔTrbE10) and *Nkx2-5*^*ΔTrbE14*^ (ΔTrbE14) mutant mice. Stars indicate the papillary muscles, and arrowheads indicate excessive trabeculations. RV: right ventricle; IVS: interventricular septum; LV: left ventricle. Scale bar = 1mm. (B) Eosin staining on transversal sections of the mid-ventricle from control, *Nkx2-5*^*ΔTrbE10*^ and *Nkx2-5*^*ΔTrbE14*^ adult hearts. The dotted lines delimit the boundary between trabecular and compact zones in LV. (C) Immunofluorescence with endoglin, SMA and VEGFR2 antibodies to delineate endocardium (Eng^high^/VEGFR2^-^) and vascular endothelial cells (Eng^low^/VEGFR2^+^) on transversal sections of the mid-ventricle from control, *Nkx2-5*^*ΔTrbE10*^, and *Nkx2-5*^*ΔTrbE14*^ adult hearts. Scale bar = 500μm. On the right panel, high magnifications of the left ventricular lumen show the numerous endocardial islets detected in *Nkx2-5*^*ΔTrbE10*^ and *Nkx2-5*^*ΔTrbE14*^ mutants (arrows). Scale bar = 100μm. (D) Perfusion of WGA-Cy3 lectin in the ventricle of control and *Nkx2-5*^*ΔTrbE10*^ hearts. (E) Immunofluorescence with endoglin, VEGFR2 and perfused WGA to detect the endocardium. Scale bar = 1mm. Below, high magnification of Eng+/VEGFR2- endocardial islets stained with perfused WGA-Cy3 (arrows). Scale bar = 100μm. (F-G) Quantification of the degree of excessive trabeculations by measuring the total length of the endocardium layer. These measures were performed for 3 sections at the level of the apex and at mid-ventricle from control (N = 3), *Nkx2-5*^*ΔTrbE10*^ (** p < 0,005, N = 3) and *Nkx2-5*^*ΔTrbE14*^ (*p < 0,05, N = 3) adult hearts (F), and counting the number of endocardial islets per section (G) (**p < 0,002, N = 3 for *Nkx2-5*^*ΔTrbE10*^ *vs* control and ^$^ p < 0,02, N = 3 *vs Nkx2-5*^*ΔTrbE14*^).

Morphological analyses using high-resolution cine-MRI showed that the papillary muscles fail to coalesce and are enlarged and separated into multiple strands in adult *Nkx2-5*^*ΔTrbE10*^ and *Nkx2-5*^*ΔTrbE14*^ hearts (Figs 2A, S1A and S1B). In addition numerous trabeculations were observed in the left ventricular cavities of *Nkx2-5*^*ΔTrbE10*^ and to a lesser extent *Nkx2-5*^*ΔTrbE14*^ hearts (Fig 2A). We confirmed these results by histological analysis on transverse sections of adult hearts and showed that the compact zone of the myocardium is not affected, nor is the right ventricle (Figs 2B and S1C). To identify ventricular trabeculations, we performed co-immunofluorescence using antibodies labeling endothelial cells, Endoglin (Eng) and VEGFR2, to distinguish Eng^+^/VEGFR2^-^ endocardium from Eng^low^/VEGFR2^+^ coronary vascular endothelial cells [23]. Excessive trabeculations evidenced by extensive invaginations of the endocardium (arrows in Fig 2C) are found in both *Nkx2-5*^*ΔTrbE10*^ and *Nkx2-5*^*ΔTrbE14*^ compared to control hearts. Four-chamber views of these hearts reveal a distribution of these trabeculations throughout the base-apex axis (Figs 2D and S1). To quantify these hypertrabeculated phenotypes, we measured the length of the endocardium in sections through the cardiac apex and at the mid-ventricular level. Endocardial length was greater in *Nkx2-5*^*ΔTrbE10*^ and *Nkx2-5*^*ΔTrbE14*^ compared to control hearts (Fig 2F). As the formation of coronary vasculature accompanies postnatal ventricular compaction [24], we investigated coronary artery density by counting the number of arteries identified by Cx40-RFP and SMA staining and observed no significant differences in artery density between these mice (S1C Fig). However, we observed the presence of endocardial cells defined by a strong expression of Endoglin (arrows) in the subendocardial myocardium (Figs 2C and S1C). These endocardial cells are organized in islets, are negative for VEGFR2 and are not associated with SMCs. Ex-vivo perfusion of the fluorescent lectin WGA-Cy3 (Wheat Germ Agglutinin) into the left ventricle of control and *Nkx2-5*^*ΔTrbE10*^ hearts demonstrated that endocardial islets are in direct communication with the endocardium, in contrast to WGA-negative coronary vasculature (Fig 2D and 2E). These results strongly suggest that endocardial islets correspond to deep endocardial invaginations due to defective trabecular compaction. Quantification revealed a significantly larger number of endocardial islets in *Nkx2-5*^*ΔTrbE10*^ mutant hearts than in control or *Nkx2-5*^*ΔTrbE14*^ hearts (Fig 2G). Together, these results suggest that excessive trabeculations detected in *Nkx2-5* mutant hearts result from defects in trabecular compaction or coalescence. Furthermore, deletion of *Nkx2-5* in embryonic *versus* fetal trabeculae results in increased phenotypic severity.

### Hypertrabeculation after conditional *Nkx2-5* loss of function is accompanied by conduction system hypoplasia and subendocardial fibrosis

**Fig 3:**
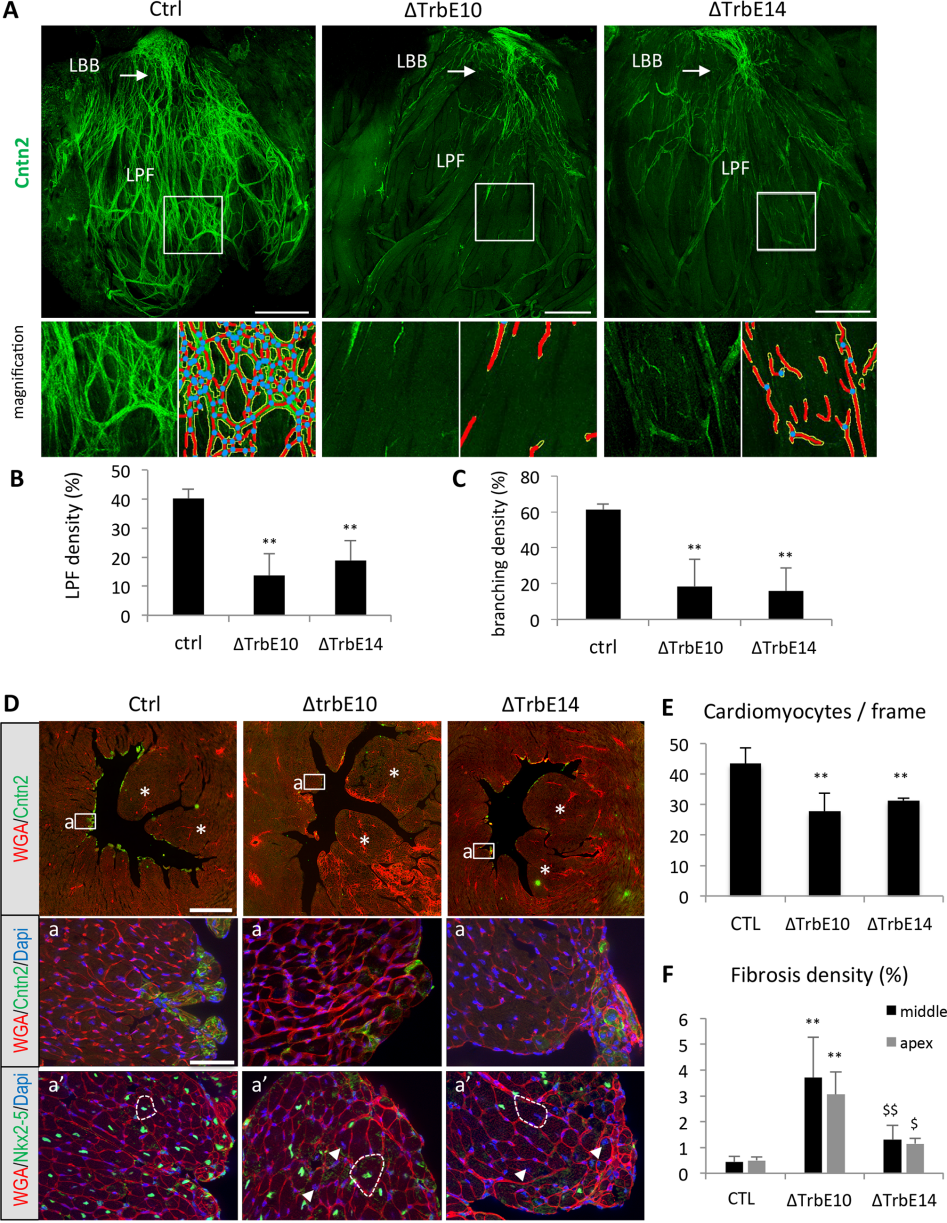
Ventricular conduction system defects, fibrosis and cardiac hypertrophy associated with *Nkx2-5*-induced hypertrabeculation. (A) Whole-mount immunofluorescence with Contactin-2 on opened LV from control (Ctrl), *Nkx2-5*^*ΔTrbE10*^ (∆TrbE10) and *Nkx2-5*^*ΔTrbE14*^ (∆TrbE14) adult hearts. High magnifications of peripheral PFs are shown below beside the corresponding images treated with angiotool. Red lines indicate the PF, and blue dots the branch points. LBB: Left bundle branch; LFP: Left PFs. Scale bar = 1mm. (B-C) Quantification of PF (B) and branching density (C). (N = 3 hearts per group) Mean ± SD; **p<0.01 *Nkx2-5*^*ΔTrbE10*^ *vs* control, **p<0.01 *Nkx2-5*^*ΔTrbE14*^ *vs* control. (D) Immunofluorescence with Contactin-2 and WGA-cy3 antibodies on transversal sections at the mid-ventricular level from control (Ctrl), *Nkx2-5*^*ΔTrbE10*^ (∆TrbE10) and *Nkx2-5*^*ΔTrbE14*^ (∆TrbE14) adult hearts. Asterisks indicate papillary muscles. Scale bar = 1mm. (a-a’) High magnifications at the level of the interventricular septum (a) and a serial section stained with WGA-cy3 and Nkx2-5 (a’). Dotted lines indicate the size of cardiomyocytes and arrowheads Nkx2-5^-^ nuclei present only in *Nkx2-5*^*ΔTrbE10*^ and *Nkx2-5*^*ΔTrbE14*^ mutants hearts. Scale bar = 50μm. (E) Quantification of cardiomyocytes hypertrophy by counting the number of cardiomyocytes per frame on high magnification images of transversal sections. (n = 20–30 frames per heart; N = 3 mice per group). **p<0.002 *Nkx2-5*^*ΔTrbE10*^ and *Nkx2-5*^*ΔTrbE14*^ *vs* control. (F) Fibrosis was quantified by measuring the percentage of WGA positive area in the LV. These measures were performed for 3 sections of adult hearts at the level of the apex and at mid-ventricle from control (N = 3), *Nkx2-5*^*ΔTrbE10*^ (**p < 0,005 (apex) and **p< 0,002 (mid) *vs* control, N = 3) and *Nkx2-5*^*ΔTrbE14*^ ($ p < 0,02 (apex) and ^$$^ p < 0,01 (mid) *vs Nkx2-5*^*ΔTrbE10*^, N = 3).

We investigated ventricular conduction system (VCS) development after conditional *Nkx2-5* inactivation, by whole-mount immunofluorescence using the VCS marker Contactin-2 (Cntn-2) in opened LV preparations of adult control, *Nkx2-5*^*ΔTrbE10*^ and *Nkx2-5*^*ΔTrbE4*^ hearts [25]. In control hearts, Cntn-2 is detected in the entire VCS including the atrioventricular bundle (AVB), left bundle branch (LBB) and Purkinje fibers (PF) network (Fig 3A). In mutant hearts after *Nkx2-5* deletion at embryonic or fetal stages, the PF network is strongly reduced while the LBB and AVB develop normally (Figs 3A and S2). We used the software “Angiotool” to quantify the complexity of the PF network [26]. In a branched structure, such as a vascular tree or the PF network, this image analysis software calculates the number of branching events as well as the length and density of vessels/fibers. Using Angiotool we observed a marked reduction of PF density and branching in *Nkx2-5* mutant compared to control hearts (Fig 3B and 3C). However, while the PF network appears less affected in *Nkx2-5*^*ΔTrbE14*^ compared to *Nkx2-5*^*ΔTrbE10*^ mutants (Fig 3A), this difference was not scored as significant using Angiotool.

In order to study the *Nkx2-5* conditional mutant cardiac phenotype in more detail, we performed immunofluorescence on transverse sections using WGA, a lectin used to quantify cardiac fibrosis and cardiomyocyte size [27]. Extensive subendocardial fibrosis was detected in *Nkx2-5*^*ΔTrbE10*^ and *Nkx2-5*^*ΔTrbE14*^ hearts while no evident signs of fibrosis were observed in control mice (Figs 3D, S1D and S3). Interstitial fibrosis was quantified by measuring the percentage of fibrotic area in the LV and revealed a more extended fibrosis in *Nkx2-5*^*ΔTrbE10*^ than *Nkx2-5*^*ΔTrbE14*^ mutant hearts (Fig 3F). WGA staining also revealed a hypertrophic phenotype in *Nkx2-5*^*ΔTrbE10*^ and *Nkx2-5*^*ΔTrbE14*^ mutants (Fig 3D-a,a’). The number of cardiomyocytes/field is significantly reduced in these two mutants compared to control (Fig 3E). This hypertrophic phenotype is independent of Nkx2-5 expression as it was observed in both Nkx2-5-positive and Nkx2-5-negative, *i*.*e*. non-deleted as well as deleted cardiomyocytes (arrowheads in Fig 3D-a’).

In summary, anatomical and morphological criteria demonstrate that conditional *Nkx2-5* loss of function in *Cx40* expressing trabecular myocardium results in excessive trabeculations. This phenotype is accompanied by trabecular compaction defects including absence of papillary muscle coalescence, deep protrusions of Endoglin^+^ cells resulting in endocardial islets, hypoplasia of the PF network, extensive subendocardial fibrosis and cardiac hypertrophy. Moreover, distinct severe and mild phenotypes were observed following *Nkx2-5* deletion using *Cx40-cre* at embryonic and fetal stages respectively, suggesting a relationship with the extent of trabeculae-derived myocardium in which *Nkx2-5* has been deleted.

### Hypertrabeculation is associated with an upregulation of cardiac proliferation without disturbing the compact-trabecular boundary at fetal stages

**Fig 4:**
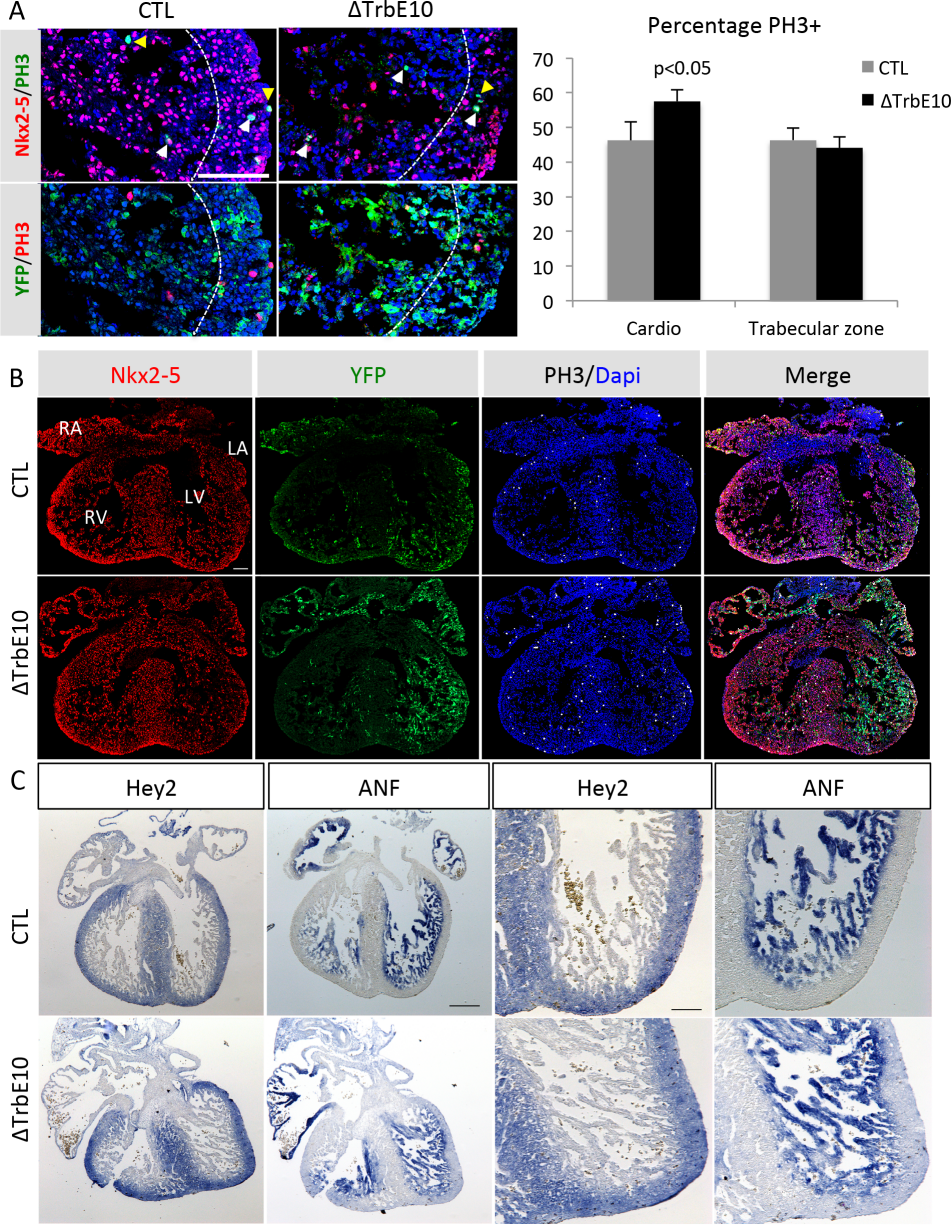
Analysis of proliferation and the compact trabecular boundary in fetal mutant hearts. (A-B) PhosphoHistoneH3/Nkx2-5/YFP immunofluorescence of left ventricular cardiomyocytes from E14.5 control and *Nkx2-5*^*ΔTrbE10*^ (∆TrbE10) hearts. White and yellow arrows indicate PH3+ cardiomyocytes and non-cardiomyocytes, respectively. The boundary between the trabecular and compact zones is indicated by a dotted line. The graph shows the percentage of PH3+ cardiomyocytes per section and the percentage of these PH3+ cardiomyocytes in the trabecular zone. Quantification was performed on 3–4 sections per heart (N = 3); *p<0.05 control *vs Nkx2-5*^*ΔTrbE10*^. Scale bar = 100μm. (C) *In situ* hybridization using *ANF* (*Nppa*) and *Hey2* riboprobes on left ventricular cardiomyocytes of E14.5 control and *Nkx2-5*^*ΔTrbE10*^ (∆TrbE10) fetal hearts. Scale bar = 200μm. Right: high magnifications of left ventricles. Scale bar = 100μm.

To investigate the embryonic basis of the hypertrabeculated phenotype, we analyzed cardiomyocyte mitotic activity in the left ventricle of E14.5 control and *Nkx2-5*^*ΔTrbE10*^ hearts by PH3 immunofluorescence on sections (Fig 4A and 4B). Quantification revealed a slight excess of PH3+ nuclei in cardiomyocytes of mutant hearts, which is not restricted to the trabecular zone, suggesting a global increase of proliferation in ventricular cardiomyocytes. Using *Cx40-Cre*::*R26-YFP* genetic tracing of the same fetal hearts, we observed no difference in the distribution of YFP+ cells in mutants in either atria and ventricles (Fig 4B). Moreover, *in situ* hybridization using probes specific for the compact and trabecular zones, *Hey2* and *ANF (Nppa)* respectively, revealed the formation of a clear boundary in both E14.5 control and *Nkx2-5*^*ΔTrbE10*^ hearts (Fig 4C). The compact-trabecular transition at fetal stages is thus unaffected in mutant hearts, suggesting that the excessive trabeculation results from both defects in cardiac proliferation and impaired ventricular compaction.

### Hypertrabeculation after conditional *Nkx2-5* loss of function is associated with conduction defects and progressive HF correlated with strain defects

The above results show that conditional *Nkx2-5* loss of function using *Cx40-cre* results in new mouse models of severe and mild ventricular non-compaction. We next carried out a longitudinal study of the same animals in order to document the evolution of the hypertrabeculated phenotypes with age and the consequence of these morphological defects on cardiac function. A follow up of cardiac morphology and function was performed in individual mice over a year using cardiac MRI, echocardiography or ECG recordings.

**Fig 5:**
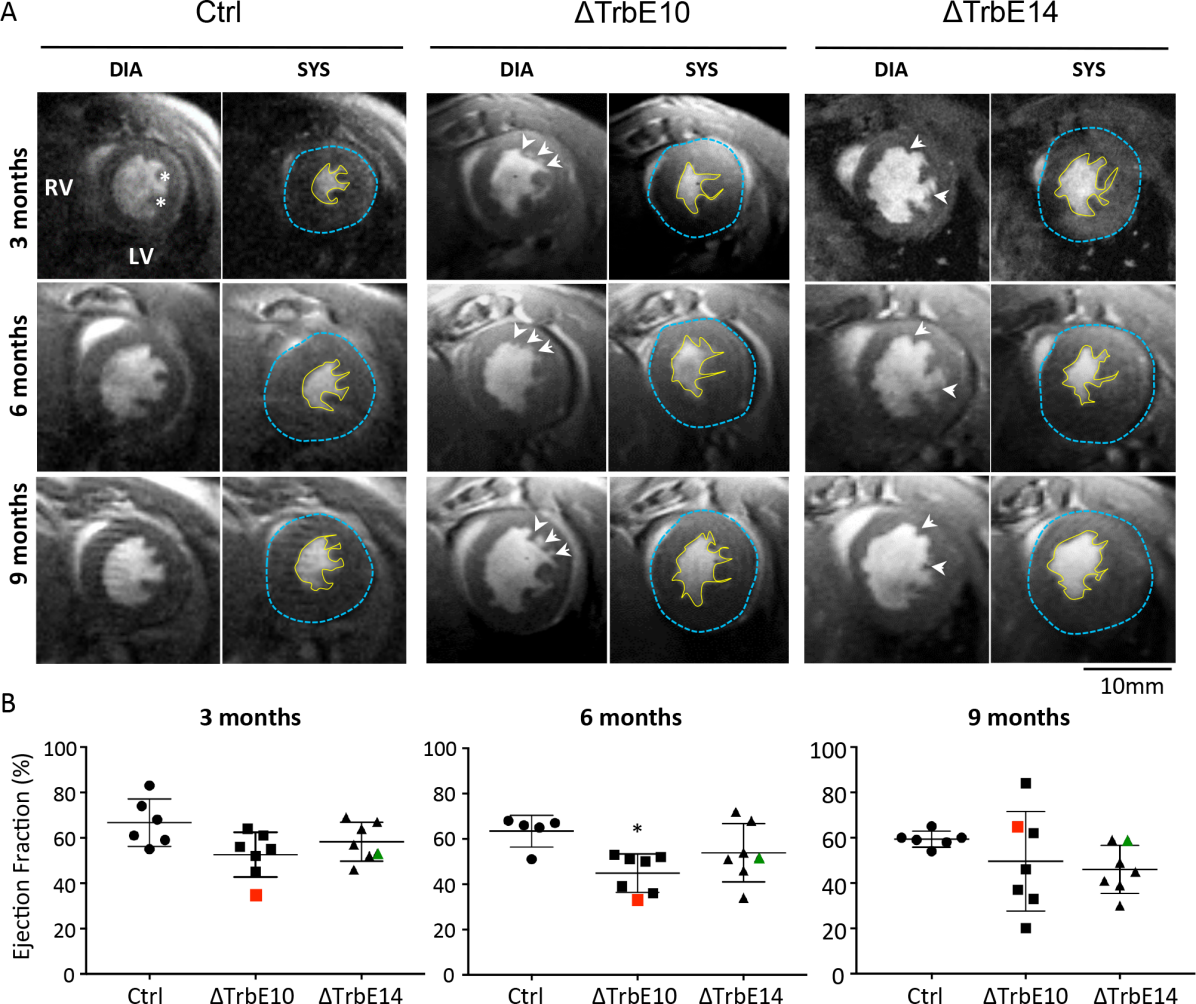
Longitudinal study of cardiac function by magnetic resonance imaging in *Nkx2-5* conditional mutant mice. (A) Short-axis cine images recorded by MRI at end-diastole (DIA) and end-systole (SYS). Images represent the follow-up of the same heart per mutant groups at 2–4, 6–8 and 9–10 month-old, with the yellow line indicating the endocardial outline and the blue line indicating the epicardial outline. Signs of hypertrabeculation are highlighted by arrows, and papillary muscles by stars. (B) Boxplot graphs represent the evolution of the ejection fraction (EF) measured in the same mice over a year. The EF corresponding to the LV capacity of contraction, was estimated by manually volumetric measurements from cine long and short axis images. Each dot represents an individual mouse. Red and green dots represent mice associated with contraction defects and fluctuating EF over time. (N = 6 in control and N = 7 for *Nkx2-5*^*ΔTrbE10*^ and *Nkx2-5*^*ΔTrbE14*^). Defects in six-month old *Nkx2-5*^*ΔTrbE10*^ were significant *vs* control, *p = 0,0165.

In order to compare trabecular morphology in *Nkx2-5*^*ΔTrbE10*^ and *Nkx2-5*^*ΔTrbE14*^ mutants, we used high-resolution cine-MRI, a highly sensitive non-invasive imaging technique. High-resolution cine-MR images revealed the presence of trabeculations in the LV of *Nkx2-5*^*ΔTrbE10*^ and *Nkx2-5*^*ΔTrbE14*^ mutants compared to the smooth endocardium observed in control hearts (Fig 5A). These results are consistent with our histological findings (Figs 2B and S1). Follow-up analysis of the same animals from 3 to 10 month-old showed indistinguishable cardiac structures at all timepoints in term of number and size of papillary muscles or trabeculations, suggesting no morphological evolution of the hypertrabeculated phenotype with age. However, several cardiac parameters measured from these MR images showed significant differences between groups and/or age (Table 1). In the line with cardiac cell hypertrophy observed in *Nkx2-5*^*ΔTrbE10*^ and *Nkx2-5*^*ΔTrbE14*^ mutants, LV mass is increased, particularly in *Nkx2-5*^*ΔTrbE10*^ hearts. At the functional levels the stroke volume is lower and the ejection fraction (EF) is reduced in *Nkx2-5*^*ΔTrbE10*^ and *Nkx2-5*^*ΔTrbE14*^ compared to control hearts revealing serious contractility defects (Fig 5B and Table 1). Interestingly, the individual follow-up showed that several mutants exhibited fluctuating EF with age (red and green dots in Figs 5B and S4); for example, an EF measured at 35% at 3 months, 33% at 6 and increased to 62% at 9 months in the same *Nkx2-5*^*ΔTrbE10*^ mouse. Movies created from short-axis cine series of these different hearts show irregular deformations of the LV during cardiac cycles and reveal that these defects are aggravated with age, revealing problems of contractility in these mutants (S1–S4 Movies).

**Table 1:** Characteristics and volumetric measurements from MRI.

	2–3 month-old			6–8 month-old			9–10 month-old			2-way Anova	
Groups	Ctrl	ΔTrbE10	ΔTrbE14	Ctrl	ΔTrbE10	ΔTrbE14	Ctrl	ΔTrbE10	ΔTrbE14	Age	Group
***Physiological parameters***											
N (males/ females)	6 (5/1)	7 (7/0)	7 (4/3)	5 (4/1)	7 (7/0)	7 (4/3)	6 (5/1)	7 (7/0)	7 (4/3)		
Body weight (g)	39,8 ± 3,6	39 ± 2,2	38 ± 2,9	45,7 ± 2,6	48,6 ± 2,6	42,4 ± 2,5	48,3 ± 3,3	54,3 ± 3,8 aa	46,4 ± 3,1	¶¶¶	NS
***Morphological parameters***											
EDV (μl)	61,2 ± 5,8	63,3 ± 2,1	50,1 ± 3,5	71,3 ± 6,9	64,4 ± 2,3	62,8 ± 9,3	75,5 ± 4	76,5 ± 7,7	69,7 ± 8,0	¶	NS
ESV (μl)	17,7 ± 3,9	23,0 ± 2,5	17,7 ± 3,0	21,5 ± 3,6	30,0 ± 3,0	25,2 ± 7,1	24,7 ± 2,8	34,5 ± 8,4	32,5 ± 5,8	¶	NS
LVmass (mg)	112,2 ± 6,0	132,2 ± 10,6	105 ± 5,1	118,5 ± 8,8	153,6 ± 17,5	125,4 ± 14,4	124,9 ± 10,3	177,7 ± 14,3*;^a^	136,6 ± 14,3	¶	¶¶
sWTn (%)	46,8 ± 6,2	34,9 ± 4,3	36,1 ± 3,5	43,2 ± 3,9	31,7 ± 6,7	40,4 ± 5,1	47,7 ± 5,1	33,4 ± 5,9	31,6 ± 4,7	NS	¶
***Functional parameters***											
EF (%)	66,7 ± 4,3	52,6 ± 3,7	58,3 ± 3,2	63,4 ± 3,1	44,9 ± 3,2	53,9 ± 4,9	59,3 ± 1,5	49,6 ± 8,3	46,0 ± 4,0	NS	¶¶
SV (μl)	43,5 ± 3,0	40,2 ± 2,4	32,4 ± 1,0	49,8 ± 4,8	34,4 ± 2,6 **	37,5 ± 3,1	50,8 ± 3,3	42,0 ± 2,0	37,2 ± 3,3 *	NS	¶¶¶¶
***Normalized morphological parameters***											
EDV (μl/g)	1,56 ± 0,15	1,65 ± 0,10	1,34 ± 0,11	1,52 ± 0,16	1,36 ± 0,11	1,47 ± 0,17	1,57 ± 0,04	1,45 ± 0,17	1,48 ± 0,08	NS	NS
ESV (μ/g)	0,45 ± 0,11	0,62 ± 0,09	0,47 ± 0,09	0,46 ± 0,08	0,62 ± 0,07	0,58 ± 0,15	0,51 ± 0,05	0,66 ± 0,16	0,68 ± 0,08	NS	NS
LVmass (mg/g)	2,89 ± 0,22	3,46 ± 0,36	2,84 ± 0,22	2,51 ± 0,15	3,21 ± 0,39	2,92 ± 0,23	2,59 ± 0,15	3,32 ± 0,29	2,90 ± 0,13	NS	¶

**N** = number of animals used in this study. **ESV** = End-Systolic Volume and **EDV** = End-Diastolic Volume, represent the internal volume of the left ventricle chamber at the end of the systole or diastole; **LV mass dia** and **LV mass sys**, represent the mass of the left ventricular wall at the end of the diastole or systole; **sWTn** = systole Wall Thickening, represents the thickening of the left ventricular wall at the end of the systole compare to the end of the diastole; **EF** = Ejection Fraction, represents the percentage of volume measured at the end of the diastole, expulsed at the end of the systole; **SV** = Stroke Volume, represent the volume of blood expulsed at the end of the systole. Data are expressed in Mean ± SE

*P<0,05 *vs* control; **P<0,01 *vs* control; ^a^P<0,05 *vs* 3 months; ^¶^P<0,05; ^¶¶^P<0,01; ^¶¶¶^P<0,001; ^¶¶¶¶^P<0,0001.

**Fig 6:**
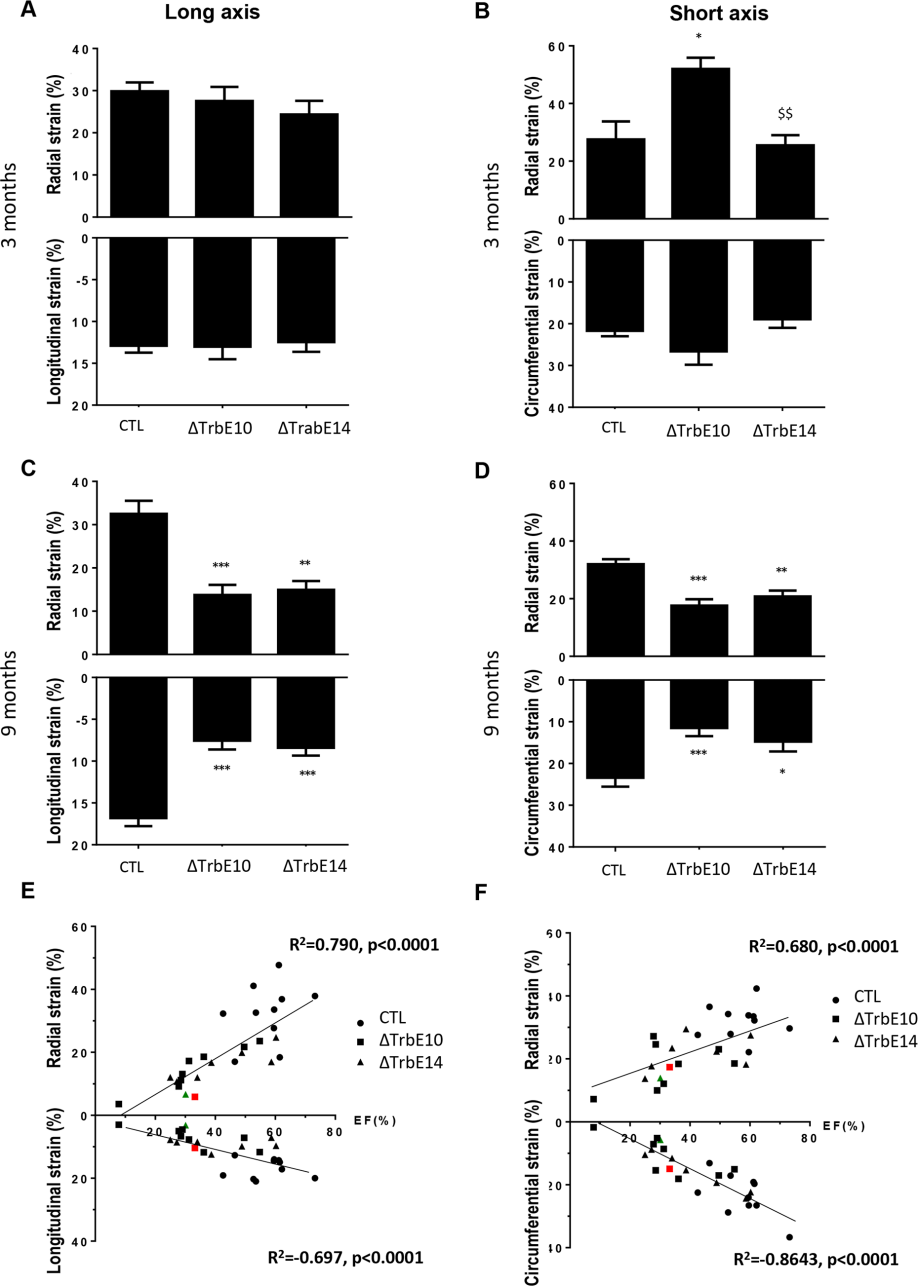
Early and late strain defects in *Nkx2-5* mutant hearts evaluated by high resolution echocardiography. (A) Histogram showing that long-axis radial and longitudinal strains are similar in 3-month-old mice. (B) The short-axis radial strain is increased in 3-month-old *Nkx2-5*^*ΔTrbE10*^ mice compared to control and *Nkx2-5*^*Δcomp*^ mice. (*p<0.01 *Nkx2-5*^*ΔTrbE10*^ *vs* control, ^$$^ p<0.01 *Nkx2-5*^*ΔTrbE10*^ *vs Nkx2-5*^*Δcomp*^; N = 6). (C-D) Long and short-axis strain parameters were decreased in 9-month-old *Nkx2-5* mutant mice compared to control (***p<0.001 *Nkx2-5*^*ΔTrbE10*^ and *Nkx2-5*^*Δcomp*^ *vs* control; N = 8–10 per groups). (E-F) Plots of Ejection Fraction (EF) against strain data recorded during long axis (E) and short axis (F) from 9-month old mice. Linear relationships are observed and R^2^ and p-values are indicated for each graph. Red and green dots represent mice with a fluctuating EF associated with strain defects.

To better appreciate the myocardial deformation, we carried out speckle-tracking based strain imaging analysis by echocardiography. At 3 months, longitudinal and radial strain measurements in the long axis were identical in all mice while radial and circumferential strain measurements in the short axis were elevated in *Nkx2-5*^*ΔTrbE10*^ hearts compared to control or *Nkx2-5*^*ΔTrbE14*^ mutants (Fig 6A). In contrast, both *Nkx2-5*^*ΔTrbE10*^ and *Nkx2-5*^*ΔTrbE14*^ mutants presented a decreased strain in all axes in old mice compared to age-matched control animals (Fig 6B). These results highlight the defects of myocardial deformation, already detected in young mice when the hypertrabeculation phenotype is most severe. In addition, the variation of strain with age is consistent with the variability of EF measured in both mutants. Interestingly, a number of mice with a severe or mild hypertrabeculation phenotype, presented signs of HF with an EF<40% suggesting that altered cardiac function is independent of the level of excessive trabeculation. Secondary to the HF phenotype, RV dysfunction was detected in 9 month-old *Nkx2-5*^*ΔTrbE10*^ and *Nkx2-5*^*ΔTrbE14*^ mice but not in 3 month-old mice (S5 Fig). Moreover, both mutants presented a reduced EF in old mice which is highly correlated with strain in all axes supporting the fact that strain is a good parameter to estimate cardiac function and HF (Fig 6C).

**Fig 7:**
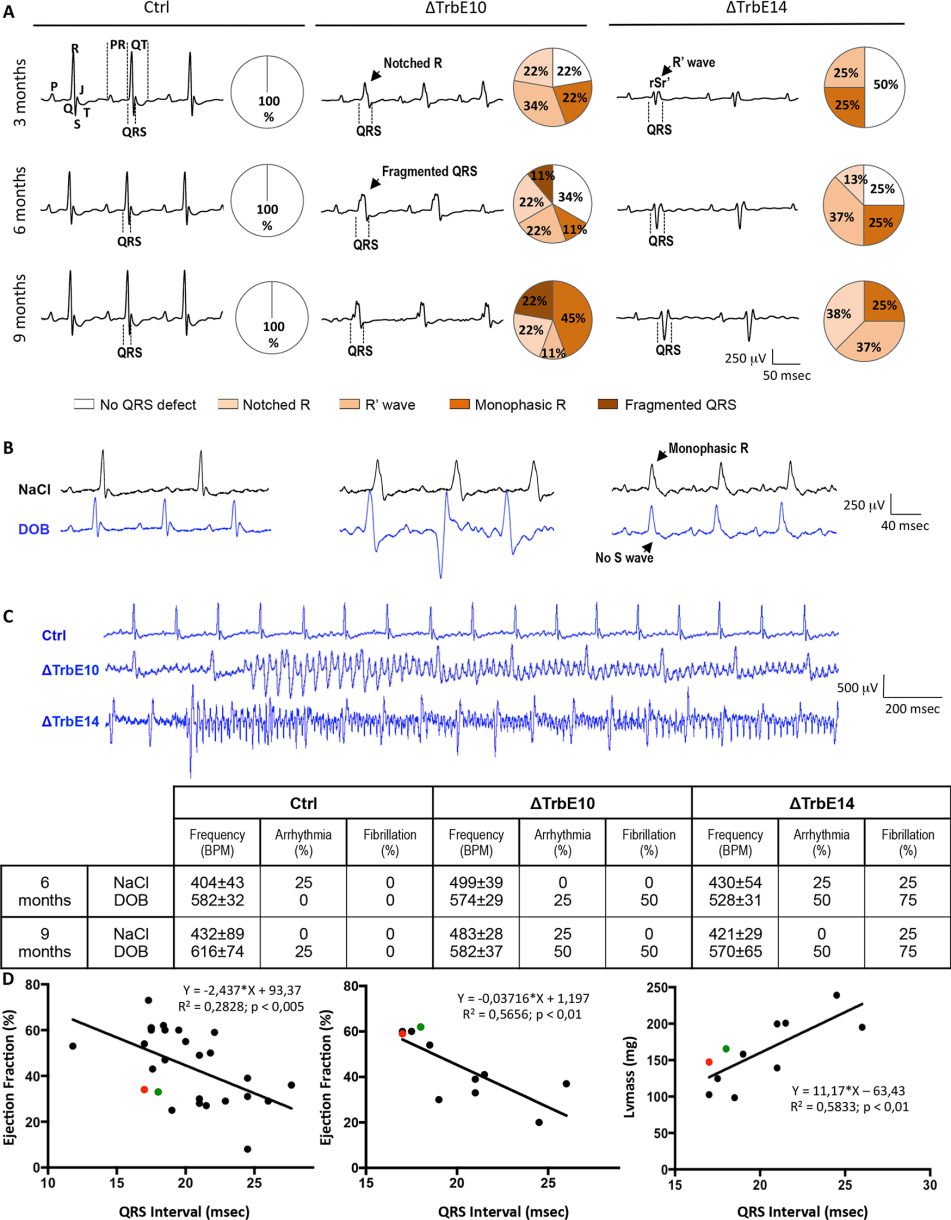
Hypertrabeculated *Nkx2-5* mutant hearts exhibit QRS defects, BB blocks and high susceptibility to fibrillation. (A) Representative tracings from surface ECG measured in lead II in anaesthetized mice at 3-, 6- and 9-month old. In control mice, QRS complexes are normal regardless of the age at which they were recorded. Tracings from *Nkx2-5*^*ΔTrbE10*^ and *Nkx2-5*^*ΔTrbE14*^ mice demonstrated bundle branch block-like appearance with widened QRS complex and a characteristic pattern with Notched R, R' wave and fragmented QRS. (B) Dobutamine caused a marked increase of heart rate in control mice and only a small effect in *Nkx2-5*^*ΔTrbE10*^ and *Nkx2-5*^*ΔTrbE14*^ mice. (C) Severe susceptibility to ventricular fibrillation was found in *Nkx2-5*^*ΔTrbE10*^ and *Nkx2-5*^*ΔTrbE14*^ mutants but not in control mice. Cardiac frequency and quantification of arrhythmias and fibrillation before and after Dobutamine injection are summarized in the table. (D) Plots of QRS duration in lead II from 9-month old mice against Ejection Fraction (EF) recorded during echocardiography (N = 27) and MRI (N = 11) or against LV mass from MRI (N = 11). Red and green dots represent mice with fluctuating EF overtime. Linear relationships were observed and R^2^ and p-values were indicated for each graph.

In parallel, we performed a longitudinal study in the same group of mice to investigate the cardiac electrical activity by measuring surface electrocardiograms (ECGs). The analysis of ECG traces revealed major defects in the QRS complex from 3 month-old in *Nkx2-5*^*ΔTrbE10*^ and *Nkx2-5*^*ΔTrbE14*^ mutants (Fig 7A). Monophasic R and R' waves were early observed in both mutants and Notched R wave was first detected in *Nkx2-5*^*ΔTrbE10*^ mutant mice. Fragmented QRS, the more drastic defect, was only recorded in *Nkx2-5*^*ΔTrbE10*^ mutant mice (Fig 7A). At 9 months, none of the *Nkx2-5* mutants had a normal QRS in contrast with control mice that never showed QRS defects. Analysis of time intervals and wave amplitudes is summarized in Table 2. Consistent with observations of QRS shape, broader QRS intervals with smaller amplitude of R waves were found in *Nkx2-5*^*ΔTrbE10*^ mutant and to a lesser extent in *Nkx2-5*^*ΔTrbE14*^. These results illustrate bundle branch blocks (BBB) in both mutants. Moreover, QRS intervals duration correlates fully with EF, measured by cardiac MRI and echography, and LV mass (Fig 7D). Indeed, animals with the lowest EF had the broadest QRS intervals. Note that only cardiac activation was disturbed. The repolarization phase, represented by QT intervals and T wave amplitude, was not impacted (Fig 7A and Table 2). Moreover, increased PR intervals, indicative of 1^st^ degree atrioventricular block in accordance with the atrial deletion of *Nkx2-5*, were evidenced as previously reported in *Nkx2-5* mutants [10, 28]. Because ECGs were recorded under anesthesia, the β1-agonist dobutamine (DOB) was used to challenge the hearts. Heart rate was identical in all untreated animals (Table 2), and increased after treatment by 45 and 47% at 6 and 9 months respectively, in control. In contrast, *Nkx2-5*^*ΔTrbE10*^ and *Nkx2-5*^*ΔTrbE14*^ mutants displayed chronotropic incompetence after DOB injection; heart rate increased by only 15–21% and 25–36% (in 6–9 month-old mice respectively) in line with a decreased cardiac reserve particularly in *Nkx2-5*^*ΔTrbE10*^ (Fig 7B and 7C). However, arrhythmia and a severe susceptibility to ventricular fibrillation were unveiled in both mutants but not in control mice (Fig 7C).

**Table 2:** Surface ECG parameters.

	3 month-old			6 month-old			9 month-old			2-way Anova	
Groups	Ctrl	ΔTrbE10	ΔTrbE14	Ctrl	ΔTrbE10	ΔTrbE14	Ctrl	ΔTrbE10	ΔTrbE14	Age	Group
N (males/females)	10	9	8	9	9	8	10	9	8		
Heart Rate (BPM)	531 ± 34	481 ± 23	503 ± 23	486 ± 25	490 ± 28	451 ± 19	498 ± 26	509 ± 14	459 ± 24	NS	NS
PR (msec)	33,1 ± 0,7	36,9 ± 1,1	32,3 ± 1,2	31,8 ± 1,0	39,9 ± 1,6 ***	36,7 ± 2,0	34,2 ± 1,2	38,3 ± 1,3	36,9 ± 1,2	NS	¶¶¶¶
QRS (msec)	17,7 ± 0,2	21,1 ± 0,9 **	19,2 ± 0,8	17,7 ± 0,4	20,8 ± 1,2 *	19,8 ± 0,7	17,7 ± 0,2	22,9 ± 1,0 ****	21,1 ± 0,8 *	NS	¶¶¶¶
QRS lead III (msec)	11,7 ± 0,3	14,2 ± 0,7 **	12,7 ± 0,3	11,9 ± 0,2	12,7 ± 0,6	13,6 ± 0,5	11,5 ± 0,3	13,8 ± 0,6 *	13,7 ± 0,7 *	NS	¶¶¶¶
QT (msec)	41,8 ± 0,6	47,4 ± 2,0	43,1 ± 1,2	44,1 ± 1,2	45,1 ± 1,2	46,0 ± 3,5	47,0 ± 2,1	51,7 ± 2,9	46,0 ± 1,7	NS	NS
T (mV)	66,1 ± 10,6	69,1 ± 8,8	96,3 ± 14,6	90,2 ± 11,9	101,7 ± 12,1	86,1 ± 15,4	88,6 ± 9,1	103,8 ± 11,1	83,1 ± 14,9	NS	NS
QRS (mV)	860,5 ± 85,6	668,3 ± 95,9	832,5 ± 126,8	872,2 ± 65,1	722,2 ± 132,2	753,1 ± 124,5	805,5 ± 70,9	603,3 ± 57,7	693,1 ± 67,0	NS	NS
T/QRS (%)	7,2 ± 0,9	11,3 ± 1,3	12,0 ± 1,8	10,1 ± 1,4	16,5 ± 2,5	11,4 ± 1,3	10,9 ± 0,7	18,1 ± 2,4 *; **a**	12,3 ± 2,3	¶	¶¶¶
S (mV)	192,5 ± 11,1	220,0 ± 68,6	223,1 ± 58,0	188,9 ± 21,7	180,6 ± 47,3	192,5 ± 42,3	155,5 ± 30,5	146,1 ± 27,7	173,8 ± 47,5	NS	NS
T/S (%)	33,4 ± 4,9	47,6 ± 9,2	69,2 ± 22,4	63,1 ± 18,2	98,7 ± 31,0	65,1 ± 17,5	83,8 ± 17,7	152,0 ± 67,1	112,8 ± 40,0	¶	NS
R (mV)	668,0 ± 77,2	448,3 ± 63,3	609,4 ± 104,7	683,3 ± 68,2	541,7 ± 91,1	560,6 ± 116,4	650,0 ± 56,4	457,2 ± 47,4	519,4 ± 85,2	NS	¶
T/R (%)	9,2 ± 1,1	16,6 ± 1,6	19,5 ± 5,8	12,7 ± 1,8	21,2 ± 2,7	17,0 ± 2,1	13,5 ± 0,6	23,7 ± 2,8	17,5 ± 3,6	NS	¶¶¶

**N** = number of animals used in this study. All parameters were recorded in lead II except for QRS in Lead III as indicated. Data are expressed in Mean ± SEM

*P<0,05 *vs* control; **P<0,01 *vs* control; ***P<0,001 *vs* control; ****P<0,0001 *vs* control; ^a^P<0,05 *vs* 3 months; ^¶^P<0,05; ^¶¶¶^P<0,001; ^¶¶¶¶^P<0,0001.

Together, these data demonstrate that severe and mild hypertrabeculation phenotypes are coupled with severe defects in cardiac activation and contractility. Hypertrabeculation associated with fibrosis, hypertrophy and Purkinje fibers hypoplasia is linked with early conduction defects, leading progressively to strain defects and HF. This cardiac phenotype is similar in both mutants, though delayed in *Nkx2-5*^*ΔTrbE14*^ mice.

### Hypertrabeculation after conditional *Nkx2-5* loss of function is supported by transcriptional changes in developmental, functional and inflammatory processes

**Fig 8:**
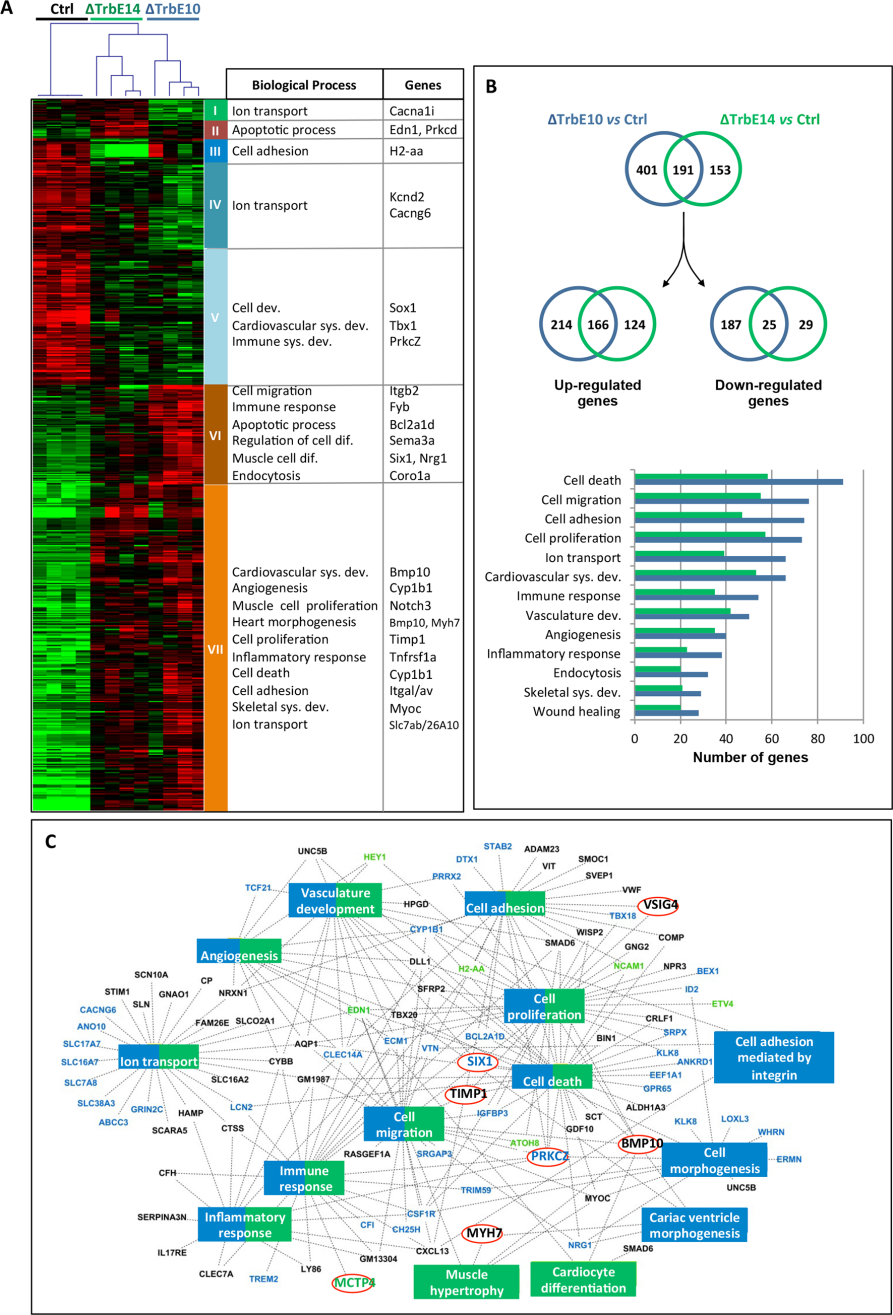
Hypertrabeculated *Nkx2-5* mutant hearts suffered transcriptional changes in developmental, functional and inflammatory processes. (A) 2D Hierarchical clustering of differentially expressed genes (Significance Analysis of Microarrays (SAM) 3 classes, 10,000 permutations, FDR < 0,05, TMeV) shows clearly the 3 differentiate groups corresponding to Control, *Nkx2-5*^*ΔTrbE10*^ and *Nkx2-5*^*ΔTrbE14*^ 6 month-old mutant mice (N = 4 per group). Enriched Biological Process (BP)-GO terms for the 7 clusters are indicated and representative genes (selected for their role in cardiomyopathies or described in Nkx2-5 mutants) are noted. (B) Venn diagrams show the number of genes significantly up- and down-regulated in *Nkx2-5*^*ΔTrbE10*^ and *Nkx2-5*^*ΔTrbE14*^ mutants *vs* control mice (SAM 2 classes, FDR < 0.05, TMeV). Note that specifically up-regulated genes are twice numerous than the down-regulated genes and that the number of genes deregulated in *Nkx2-5*^*ΔTrbE10*^ mutant is much greater than in *Nkx2-5*^*ΔTrbE14*^ mutant (592 *vs* 344). A gene ontology analysis realized with David Database is reported on the graph in which the most significant representative BP are indicated. For all processes, the number of genes deregulated in *Nkx2-5*^*ΔTrbE10*^ mutant is much greater than in *Nkx2-5*^*ΔTrbE14*^ mutant. (C) Gene network diagram carried out with venn diagram genes whose fold change is ≥ 2. Blue rectangles correspond to the most significant BP found in *Nkx2-5*^*ΔTrbE10*^ mutant and green rectangles, BP found in *Nkx2-5*^*ΔTrbE14*^ mutant. A vast majority of the most significant BP were jointly found in both mutants and are colored in blue/green (David Database, P-Benjamini <0.05). Genes implicated in these pathways are indicated in blue when they are specific for *Nkx2-5*^*ΔTrbE10*^, in green for *Nkx2-5*^*ΔTrbE14*^ mutant and in black when they are common. Red circles indicate genes validated by qPCR.

In order to identify transcriptional changes associated with hypertrabeculation and functional defects, we performed a microarray analysis on LV tissue including the ventricular free wall and apex from *Nkx2-5*^*ΔTrbE10*^, *Nkx2-5*^*ΔTrbE14*^ and control adult hearts. Comparison between both mutants and the control showed 622 differentially expressed probes, corresponding to 469 genes among the 39,430 mouse genes screened. A comprehensive list of the significantly misregulated genes is presented in a supplementary file. The hierarchical clustering in both dimensions (samples and genes) showed clear differences between the 3 groups (Fig 8A). Seven clusters, from an unsupervised hierarchical clustering based on the similarities of expression, were analyzed using Gene Ontology (GO). The most significant Biological Processes (BP) are associated with cardiovascular development, differentiation, inflammation, apoptosis and ionic transport (Fig 8A) and totally support the morphological and functional data. Major genes known for their role in LVNC or other cardiomyopathies and genes previously reported in *Nkx2-5* mutants were found to be deregulated in both mutants (Fig 8A).

Comparison of gene profiles revealed that conditional inactivation of *Nkx2-5* in ventricular trabeculae at embryonic stage resulted in the deregulation of many more genes than at fetal stage: 601 *vs* 349 respectively, 26% of which were common, as shown on the Venn diagram in Fig 8B and in the lists of the supplementary file. Specifically up-regulated genes after *Nkx2-5* deletion *vs* control were twice as numerous as down-regulated genes in *Nkx2-5*^*ΔTrbE10*^ adult hearts, 64% of which were up-regulated and 36% down-regulated. In *Nkx2-5*^*ΔTrbE14*^ hearts, 84% of them were up-regulated by the deletion of *Nkx2-5* at fetal stage while 16% were down-regulated (Fig 8B). Interestingly, GO analysis of differentially expressed genes revealed that the same biological processes were involved in both mutants but the number of genes within each process was greater in *Nkx2-5*^*ΔTrbE10*^ hearts. Therefore these data confirm that *Nkx2-5* deletion is associated with substantial transcriptional changes causing a deep remodeling of the heart, and are consistent with the observation of a stronger phenotype in *Nkx2-5*^*ΔTrbE10*^ hearts.

In order to obtain a comprehensive overview of biological processes associated with hypertrabeculation after *Nkx2-5* deletion, an integrative network was created with cytoscape (Fig 8C). While most of the BP were common to both embryonic and fetal deletions, a few pathways related to cardiac morphogenesis and cell adhesion were specific to *Nkx2-5*^*ΔTrbE10*^ mutant while *Nkx2-5*^*ΔTrbE14*^ was associated with cardiac differentiation and hypertrophy terms. Moreover, the majority of genes were transcription factors (TF) involved in a variety of developmental processes. This network illustrates clearly the impact of *Nkx2-5* deletion on fundamental processes including morphogenesis and cell differentiation.

**Fig 9:**
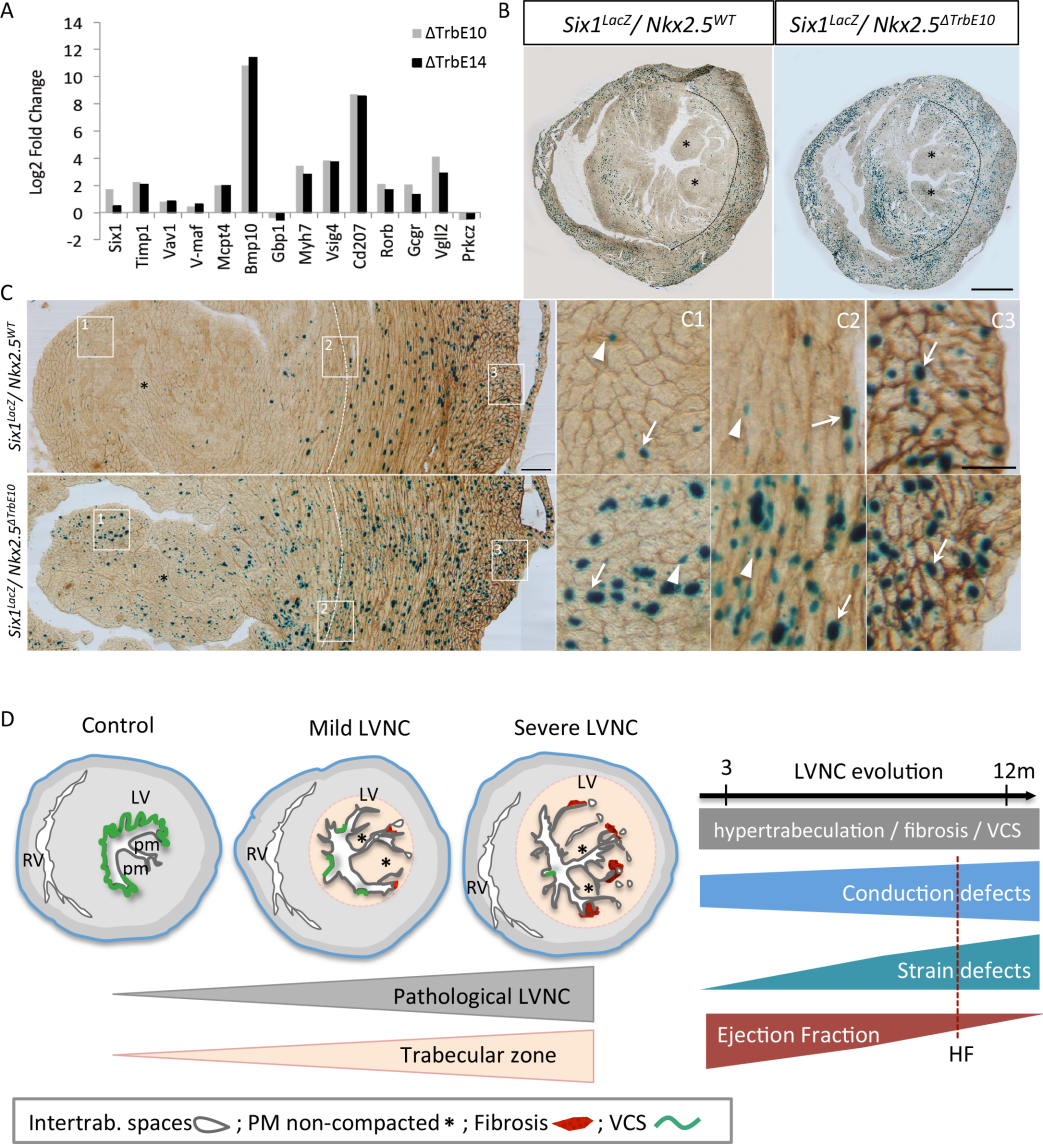
Molecular markers associated with hypertrabeculated *Nkx2-5* mutant hearts and mouse models of pathological LVNC. (A) Quantitative real-time PCR performed for a list of selected genes. The housekeeping gene used was RPL32. (B-C) X-gal (B) and Pecam-1 (C) co-immunostaining on transversal sections of the mid-ventricle from *Six1*^*LacZ/+*^::*Nkx2-5*^*+/+*^ and *Six1*^*LacZ/+*^::*Nkx2-5*^*ΔTrbE10*^ adult hearts. Dotted lines indicate the boundary between the trabecular and compact zone of the left ventricular myocardium; asterisks, the papillary muscles (PM); arrows, β-positive cardiomyocytes; arrowheads, β-gal-positive endothelial cells. (C1-C3) High magnifications of boxes at the level of the PM (C1), the trabecular/compact boundary (2) and the epicardium (3) on the LV. Scale bar B = 1mm; C = 100μm; C1-3 = 50μm. (D) Mouse models of mild and severe hypertrabeculation associated with pathological features including intertrabecular recesses, non-compacted papillary muscles, fibrosis and VCS hypoplasia. The degree of hypertrabeculation is relative to the extent of trabecular zone affected. The follow-up of cardiac function shows a correlation of impaired Ejection Fraction with conduction and strain defects in absence of aggravation of the hypertrabeculation phenotype with age. HF: Heart failure.

To validate the microarray data, we performed qPCR experiments for 14 genes that were among the most up- or down-regulated genes, selected on the basis of their cardiovascular-related function. Among the up-regulated genes in *Nkx2-5*^*ΔTrbE10*^ *vs* control, *v-Maf* and *Six1* transcripts were quantified and *Prkcz* as a down-regulated gene. *Mcpt4* was investigated as an up-regulated gene in *Nkx2-5*^*ΔTrbE14*^ *vs* control hearts. We also analyzed genes in the common part of the Venn diagram including *Bmp10*, *Timp1*, *Cd207*, *Vsig4*, *Myh7*, *Vav-1*, *Rorb*, *Gcgr* and *Vgll2* that are up-regulated in *Nkx2-5*^*ΔTrbE10*^ and *Nkx2-5*^*ΔTrbE14*^ *vs* control hearts. RT-PCR analysis confirmed the microarray results with regard to fold-changes and significance and highlighted the important differential expression of *Six1* between *Nkx2-5*^*ΔTrbE10*^ and *Nkx2-5*^*ΔTrbE14*^ mutants (Fig 9A). We further investigated this using a *Six1-LacZ* reporter line [29]. X-gal staining in the LV was restricted to the compact zone in control mice while the staining was increased and extended to the trabecular zone in *Nkx2-5*^*ΔTrbE10*^ mutant mice (Fig 9B). Pecam1 co-immunostaining revealed previously unreported *Six1* expression in endothelial cells as well as in cardiomyocytes with high density of positive cells in the compact zone of control hearts (Fig 9C1-3). In *Nkx2-5* conditional mutant hearts, an excessive number of β-gal positive cells, *i*.*e*. cardiomyocytes and endothelial cells was observed in the entire ventricular wall (Fig 9C1-3). This result validated the up-regulation of *Six1* in *Nkx2-5* loss of function hypertrabeculated hearts.

Together, our results show that deletion of *Nkx2-5* using *Cx40-cre* at embryonic and fetal stages disturbs the normal development and differentiation of the cardiovascular system. Moreover, *Nkx2-5* deletion in ventricular trabeculae provokes more molecular disturbances in adult hearts when it occurs at embryonic rather than fetal stages. Nevertheless, the same BP are affected in both cases, fully supporting the morphological and functional defects documented in these hypertrabeculated hearts. Interestingly, *Six1* expression is found in the trabecular zone only in hypertrabeculated hearts suggesting that Six1 is a potential genetic marker of LVNC.

## Discussion

In this study, we have analyzed the pathological consequences of excessive ventricular trabeculation, induced by spatio-temporal deletion of *Nkx2-5* during trabecular development, on adult cardiac function. The loss of Nkx2-5 at embryonic stages resulted in a severe hypertrabeculated phenotype associated with excessive subendocardial fibrosis, while these phenotypes were milder when deletion of *Nkx2-5* occurred at fetal stages. Our analysis revealed that the excessive trabeculation phenotype is more severe when *Nkx2-5* deletion occurs at embryonic rather than at fetal stages, consistent with progressive reduction of *Cx40-*derived trabecular cell incorporation in the myocardial wall during ventricular morphogenesis [16]. Moreover, this phenotype was stable in old mice, suggesting that the level of hypertrabeculation is associated with the broader extension of *Nkx2-5* deletion in the ventricular myocardium rather than with aging. Another feature linked to hypertrabeculation was the subendocardial fibrosis observed in *Nkx2-5* conditional mutant hearts. This result is in agreement with a recent report that subendocardial fibrosis is commonly observed in LVNC patients [30]. As in the case of hypertrabeculation, subendocardial fibrosis is more pronounced in *Nkx2-5*^*ΔTrbE10*^ compared to *Nkx2-5*^*ΔTrbE14*^ hearts. This is consistent with previous studies showing the absence of fibrosis in hearts after conditional knockout of *Nkx2-5* after mid-gestation, at perinatal or postnatal stages [31, 32]. We observed that numerous genes associated with fibrosis are specifically upregulated in the LV after embryonic deletion of *Nkx2-5* and less affected at fetal stages. Together, these data strongly suggest that subendocardial fibrosis originates from an early event due to a dysfunction of ventricular trabeculae development rather than as a consequence of aging, as has been suggested in human patients [30]. This data is of major interest as subendocardial fibrosis may contribute to altered longitudinal and axial contraction, measured in strain analysis, and to BBB and fibrillation [33–35]. Our mouse models of hypertrabeculation thus mirror the clinical situation and support the conclusion that the degree of non-compaction results primarily from developmental defects.

Ventricular compaction is a poorly understood step of cardiac morphogenesis. Using two independent genetic tracing mouse models, it has been recently demonstrated that embryonic cardiomyocytes of the trabecular or the compact zone mix in a hybrid zone, which covers a large proportion of the compact myocardium in the adult heart [36]. Abolishing proliferation in the compact zone leads to a hypertrabeculation phenotype that is not observed if proliferation is altered in the trabecular zone only [36]. These data suggest that non-compaction arises as a result of proliferation defects of cardiomyocytes in the compact zone. Several other mouse models including the ventricular *Nkx2-5*-conditional deletion support this assumption; however, most of these mutants present a very thin compact layer and are embryonic lethal [10, 13]. In contrast, in our *Nkx2-5*^*ΔTrbE10*^ and *Nkx2-5*^*ΔTrbE14*^ mutant hearts, hypertrabeculation was not associated with a reduction of the compact layer. However, we were able to reproduce excessive trabeculation by the conditional deletion of *Nkx2-5* in ventricular trabeculae demonstrating that disruption of trabecular development represents another mechanism leading to hypertrabeculation. Moreover, our data suggest that hypertrabeculation results primarily from trabecular coalescence defects. This model is supported firstly by the disruption of the molecular boundary between the trabecular and compact zone shown by *Six1* misexpression, which has been recently associated with right ventricular associated hypertrabeculation and fibrosis in other mouse mutants [37, 38]. While this compact-trabecular transition initiates normally in our mutants at fetal stages, proliferation defects may compromise late development of trabecular compaction. Secondly, the numerous endocardial islets detected in *Nkx2-5*^*ΔTrbE10*^ mutants are highly similar to the intertrabecular spaces connected to the LV observed in LVNC patients [3]. This endocardial phenotype suggests a defect in the formation of coronary vasculature from the endocardium that normally occurs at perinatal stages [24]. Moreover, concomitant to the compaction step, the acquisition of a spiral pattern of cardiomyocytes develops as a prerequisite for twisting contraction [2, 39]. Our strain analysis points out important defects in myocardium deformation in hypertrabeculated hearts suggesting that compaction defects plays a role later for the efficiency of cardiac contractions. All these results highlight the role of *Nkx2-5* in the complex modifications in ventricular morphology that take place during trabecular compaction and their requirement for normal cardiac function.

A major characteristic of our *Nkx2-5*-hypertrabulated mouse models is the occurrence of the numerous cardiac complications such as conduction blocks, contraction defects and HF, often associated with LVNC in symptomatic patients. Because mutations in *NKX2-5* are associated with a myriad of congenital heart diseases (CHD) in humans, this transcription factor has received much attention for its role in cardiac morphogenesis [20]. The atrial-specific knockout of *Nkx2-5* produced atrial hyperplasia, atrial septal defects and bradycardia [40]. In comparison, deletion of *Nkx2-5* in atrial myocardium induced by the *Cx40-creERT2* line is heterogeneous and gives rise only to a modest effect such as a slight increase of the PR interval; in particular, no atrial anatomical defects or reduced heart rate were detected in our mutant mice. Moreover, a direct role of Nkx2-5 on cardiac contractility has already been described. Indeed, reduced and irregular ventricular contractions are recorded by echocardiography in newborn mice after conditional deletion of *Nkx2-5* at E13.5 [32]. In these mouse models, Nkx2-5 perturbs calcium handling and sarcomere organization, which are known trigger to reduce contractility [10, 17, 31, 41]. In *Nkx2-5* hypertrabeculated hearts, we found numerous deregulated genes, involved in calcium signaling (*Ryr2*, *Sln* or *SERCA2)*, the contractile apparatus (*Myh7*, *Tnnt2)*, transmembrane ionic channels (*HCN1*, *HCN4*, *Scn5a*, *Cacna1g*, *Cacna1h*, *Kcne1)*, and cardiac development, including genes of the Notch pathway and its targets, (*Notch3*, *Dll1*, *Dtx1*, *Hey1*, *BMP10)* (transcriptomic analysis and qPCR experiments (S6 Fig)). These genes have been previously described to be deregulated in different *Nkx2-5* mutant mice and in some cases, including Myh7, HCN4 [32], Tnnt2 [42], Scn5a [43], Ryr2 [44] and Notch signaling [9], have been associated with LVNC. It has recently been shown that cardiac contractility may also depend directly on endothelial signaling through the NRG-1/Erbb2 pathways [45]. Indeed, Pecam-1 null mice display a slight decreased EF without morphological defects in cardiomyocytes or capillary densities but resulting from a disturbed communication between endothelial cells and cardiomyocytes. In our study, hypertrabeculated hearts present vascular defects including endocardial islets, which correlate with upregulation of Endoglin transcript levels. Indeed, LVNC patients are known to present defects in cardiac perfusion and embolic thrombosis is a pathological feature of non-compaction cardiomyopathy [46, 47]. This is consistent with the microarray data in which genes involving endothelial cells signaling or vasculature are found deregulated in mutant hearts. Finally, these changes in gene expression are likely to modulate cardiomyocyte activity that impact directly on cardiac contractility and pump function as measured by reduced strain rate in line with decreased EF. Intriguingly, we found fluctuations in strain and EF at different ages during the follow-up of the cardiac function in few hypertrabeculated hearts. Recently, an “undulating” phenotype in which the associated cardiomyopathic features change has been evocated in children with LVNC [3, 48]. For instance, a dilated and hypertrophic presentation with poor cardiac function changed to a hypertrophic, hypercontractile form of non-compaction and the final destination was dilated dysfunctional form of non-compaction with HF [49]. These observations highlight the extreme variability in contractility of hypertrabeculated hearts and argue in favor of an independent form of cardiomyopathy. The measurements of strain in LVNC patients confirm that cardiac deformation represents a valuable parameter for prognosis in cases of excessive trabeculations [50].

We documented the first longitudinal study of the conduction defects in a mouse model with hypertrabeculation. In our *Nkx2-5* hypertrabeculated hearts, a total or quasi-absence of PF is observed and these mice display early conduction defects, in particular LBBB consistent with this hypoplasia phenotype. Indeed, PF connect to the sub-endocardium at Purkinje-muscle junctions sites in humans to form the origin of myocardial activation [51]. Consistent with our results, disruption of the spatiotemporal expression of ion channels has been shown to induce defective propagation of impulse from endocardium to epicardium, resulting in BBB and leading to increased susceptibility to fibrillation [52]. However, these ventricular conduction defects worsened during aging suggesting that QRS shape and duration also depend on other parameters such as fibrosis and hypertrophy, which are known to impair conduction. To support this hypothesis, we found a correlation between LV mass and QRS duration. While the exact role of Nkx2-5 in ventricular conduction system development is unknown, numerous genetically modified mouse models have revealed the importance of this transcription factor for normal cardiac conduction at postnatal stages [8, 31, 32, 53]. Hypoplasia of the ventricular conduction system has not been reported in other mouse models of non-compaction or in human samples suggesting that this phenotype may be specific to this transcription factor [9, 54]. However, the presence of LVNC is significantly associated with a rapid deterioration of LV function and higher mortality when associated with abnormal ECG measurements [55]. Finally, we observed that hypertrabeculated hearts have impaired chronotropic competence and are susceptible to ventricular fibrillation under conditions of stress. This is consistent with the appearance in LVNC patients of complications such as exercise intolerance or sudden death linked to excessive trabeculation in athletes after extensive exercise [2, 3]. Our mouse data mirror the heterogeneity of the pathological outcomes among LVNC patients and suggest that LVNC has a higher incidence of cardiac death when associated with cardiac dysfunction and arrhythmias. Furthermore, these data highlight the importance of *Nkx2-5* in the development of the ventricular conduction system and the role of these defects in the pathological outcome of LVNC.

Our molecular analysis identified numerous genes related to cardiac dysfunction, and highlights the pleiotropic roles of Nkx2-5 during trabecular development. Among those genes, we observed differential upregulation of Six1 between the *Nkx2-5*^*ΔTrbE10*^ and *Nkx2-5*^*ΔTrbE14*^ mutants suggesting a role of this factor in the severity of the hypertrabeculated phenotype. Six1 is a transcription factor identified for its role for skeletal muscle and cardiac progenitor cell development [29, 37]. Our immunohistological analysis revealed upregulated Six1 expression in endothelial cells and cardiomyocytes of the trabecular zone, suggesting that deregulation of this gene is associated with cardiac pathology rather than being a direct target of Nkx2-5. Interestingly, Six1 has been recently found to be deregulated in cardiomyopathy induced by *Nkx2-5* point mutation or in human patients [56, 57]. Our data suggest that Six1 may be a good marker for pathological forms of excessive trabeculation in non-compaction cardiomyopathy.

### Conclusion

In conclusion, impaired trabecular compaction provokes a hypertrabeculated phenotype associated with a disturbed endocardial vasculature, VCS hypoplasia and subendocardial fibrosis (Fig 9D). Our data strongly suggest that complications associated with hypertrabeculation such as cardiac hypertrophy and HF arise progressively and may be a direct consequence of the conduction defects and abnormal cardiac contractility of hypertrabeculated myocardium. Interestingly, these mice present a progressive impaired cardiac function while excessive trabeculations are morphological identical throughout adult life, supporting the idea that this anomaly does not *per se* trigger the pathology in LVNC. This study clarifies the origin of the pathological outcomes associated with LVNC and, although further developments are clearly warranted, may provide helpful information for clinicians in the future for the diagnostic and prognostic evaluation of left ventricular non-compaction patients.

## Materials and methods

### Generation of mouse models

Animal procedures were approved by the ethics committee for animal experimentation of the French ministry (n° 01055.02). *Cx40-creERT2*, R26-YFP and *Six1-LacZ* mouse lines were genotyped as previously reported [21, 29, 58]. A conditional null allele of *Nkx2-5* was generated as described (*Nkx2-5flox)* [22]. To remove the Frt-flanked neomycin cassette present in this allele, *Nkx2-5flox* mice were bred with a ubiquitous flipase mouse line (ACTB:FLPe) [59]. The *Floxed-Nkx2-5-∆Neo* allele was used in the subsequent breedings with the *Cx40-creERT2* mouse line. To delete the *Floxed-Nkx2-5-∆Neo* gene, tamoxifen was injected intraperitoneally to pregnant females (200μl) for two consecutive days. Tamoxifen (T-5648, Sigma) was dissolved at the concentration of 20mg/ml in ethanol/sunflower oil (10/90). After tamoxifen treatment of pregnant females, newborn mice were recovered by caesarian section and given for adoption to CD1 females. Fifty mice were assigned to three groups: *Nkx2-5*^*ΔTrbE10*^ mice received tamoxifen injections at embryonic stages (E10.5 and E11.5), *Nkx2-5*^*ΔTrbE14*^ mice received tamoxifen injections at fetal stages (E13.5 and E14.5), and control mice are littermates with *Nkx2-5-∆Neo*^*fl/fl*^:*Cx40*^*+/+*^ genotype.

### Macroscopic and histological analyses

Macroscopic examination of the internal surface of the ventricles was previously described [60]. For histological studies, adult hearts were dissected, fixed for four hours in 4% paraformaldehyde (vol/vol) in PBS, washed in sucrose gradient, then embedded in OCT and cryosectioned. To quantify interstitial fibrosis, transverse sections were counterstained with wheat germ agglutinin-Cy3 (WGA-Cy3 from Sigma-Aldrich) as described previously [27]. For immunofluorescence, sections were permeabilized in PBS 1X / 0.2% Triton X100 for 20 min and incubated for 1 hour in saturation buffer (PBS 1X / 3% BSA / 0.1% Triton X100). Primary antibodies were incubated in saturation buffer overnight at 4°C. Secondary antibodies coupled to fluorescent molecules were incubated in saturation buffer and after washes, hearts were observed under a Zeiss Apotome microscope.

For whole-mount immunofluorescence, adult hearts were pinned on petri dish to expose the LV and fixed in 4% paraformaldehyde for 2 hours at 4°C, washed in PBS, permeabilized in PBS 1X / 0.5% Triton X100 for 1h and incubated for 3 hours in saturation buffer (PBS 1X / 3% BSA / 0.1% Triton X100). The primary antibodies were incubated in saturation buffer for 24 hours at 4°C. Secondary antibodies coupled to fluorescent molecules were incubated in saturation buffer and after washes, hearts were observed under a Zeiss LSM780 confocal microscope.

The measure of the endocardial length (mm) was carried out using Image J software by drawing the contour of the endocardium (Eng1+/VEGFR2-) lining the LV on transversal sections and the mean of 3 sections per heart was calculated (n = 3 mice of each group). The number of PH3+ cells per LV section was quantified using Zeiss Zen software from LSM780 confocal images. The PH3+ cardiomyocytes are stained for Nkx2-5+ or YFP+. The percentage of PH3+ cardiomyocytes over the number of PH3+ cells was calculated from the total number of cells of 3–4 sections per heart (n = 3 embryos of each group).

Antibodies used in this study are specific to Nkx2-5 (Sc8697 Santa-Cruz), GFP (AbD Serotec), RFP (Rockland), Contactin-2 (AF1714 R&D system), Pecam-1 (MEC13.3-BD Pharmingen), Endoglin CD105 (MJ7/18-DSHB), VEGFR2 (AF644-R&D SYSTEMS), α-Smooth Muscle Actin (F3777-SIGMA).

### *Ex-vivo* perfusion in the LV of the lectin WGA-Cy3

After dissection, adult hearts were rapidly intubated with a 24G Surflo IV catheter in the aortae and perfused with 1ml of PBS heparin to remove blood, then 200μl of WGA-Cy3 was introduced in the LV through the same catheter and incubated for 30 minutes before fixation in 4% PFA. After fixation, hearts are processed as described above.

### *In situ* hybridization

Non-radioactive *in situ* hybridization on sections from E14.5 hearts were performed as described [61] using Hey2 and ANF mRNA probes.

### Cardiac magnetic resonance imaging (MRI)

MRI was carried out every two months on the same animal groups from 2 to 12 months-old mice. The experiments were performed on a Bruker Biospec Avance 4.7 T/30 imager (Bruker Biospin GmbH, Ettlingen, Germany) (France Life Imaging network), as previously described [62]. Anesthesia was maintained during MRI with 1.5–2% Isoflurane in a constant flow of room air (270 ml/min) through a nose cone using a dedicated vaporizer (univentor anaesthesia unit, univentor high precision instrument Zejtun, Malta). Temperature was maintained at 39°C. Breath and heart rate were monitored and signals were used to trigger MRI acquisition using a monitoring and gating system (SA Instruments, Inc. Stony Brook, NY, USA). Cine-MRI (ECG gating, repetition time 5 ms, echo time 1.51 ms, flip angle 20°, slice thickness 1 mm, pixel size 0.195×0.195 mm^2^) were performed in short axis view. Ten phases per heartbeat were acquired from base to apex to cover the whole LV. In addition, one high resolution cine series (ECG gating, repetition time and frame rate 15 ms, echo time 1.68 ms, flip angle 30°, slice thickness 1.1 mm, pixel size 0.086×0.086 mm^2^) was acquired in short axis view at the mid base-apex location to evaluate trabecular morphology.

### Surface electrocardiography

Surface ECGs were performed on anesthetized mice. An induction with 5% isoflurane was followed by maintenance at 1 to 2% in a constant flow of oxygen at 700 ml/min. ECGs were recorded every two months from 2 to 12 months using a bipolar system in which the electrodes were placed subcutaneously at the right (negative) and left forelimb (reference) and the left hindlimb (positive) for lead II and at the right (reference) and left forelimb (negative) for lead III. Electrodes were connected to a Bioamp amplifier (AD Instruments) and were digitalized through an A/D converter ML 825 PowerLab 2/25 (AD Instruments). Digital recordings were analyzed with Chart software for windows version 5.0.2 (AD Instruments). Events were registered to 100 K/s and were filtered to 50 Hz. ECG recordings were obtained for 1 min after stabilization of the signal. Post-analysis was performed for heart rate, PR, QRS, QT intervals, T, R and S durations, T, R, S and QRS amplitudes.

A Dobutamine (DOB) stress-test was performed on selected 6 and 9 month-old mice. After recording of ECG as described above, mice received an intraperitoneal single injection of DOB at a dose of 0,75 μg/g body weight or an equivalent volume of vehicle (0,9% NaCl). The DOB dose was selected after a bibliographic study on the criteria of a moderate increase of heart rate and a preliminary experiment using the dose of 0,75 μg/g in which two volumes of injection were tested. The lowest volume was chosen and DOB was prepared as follows:

DOB hydrochloride powder (Sigma-Aldrich, product number D-0676) was dissolved in ddH_2_O and vortexed to provide a 10 mg/ml DOB stock solution. DOB working solution (0,1 μg/μl) was prepared by 1:100 dilution of the stock solution in sterile 0,9% NaCl.

### Echocardiography

Echocardiography was performed using a Vevo 2100 ultrasound system (VisualSonics, Toronto, Canada) equipped with a real-time micro-visualization scan head probe (MS-550D) operating at a frame rate ranging from 740 frames per sec (fps). Mice were anesthetized with isoflurane (IsofloH, Abbott S.A, Madrid, Spain) at a concentration of 3.5% for induction and between 1 to 1.5% for maintenance during the analysis with 100% Oxygen. Each animal was placed on a heated table in supine position with extremities attached to the table through four electrocardiographic leads. The chest was shaved using a chemical hair remover (Veet, Reckitt Benckise, Granollers, Spain). Ultrasound gel (Quick Eco-Gel, Lessa, Barcelona, Spain) was applied to the thorax surface to optimize the visibility of the cardiac chambers. The heart rate (HR) and respiratory rate of mice were recorded during the echocardiographic study. Two mice were excluded from the study due to very low HR. Echocardiograms were acquired at baseline. Left ventricular (LV) characteristics were quantified according to the standards of the American Society of Echocardiology and the Vevo 2100 Protocol-Based Measurements and Calculations guide, as described in the following paragraphs. LV diameters were measured on a two-dimensional (B-mode) parasternal long axis and short axis view. The functional parameters of the heart were evaluated based on LV diameter measurements.

### Microarrays

Total RNA was isolated from LVs (left ventricular wall with apex and without the interventricular septum) of 6 month-old control, *Nkx2-5*^*ΔTrbE10*^ and *Nkx2-5*^*ΔTrbE14*^ mice (n = 4 for each group) using TRIzol Reagent according to the manufacturer’s procedure (Invitrogen, Life Technologies). The concentration of RNA was determined by reading absorbance at 260 nm on NanoDrop (ND-1000, ThermoScientific). The quality of RNA was confirmed on the Agilent 2100 Bioanalyzer (Agilent technologies, Germany) with the Agilent RNA 6000 Nano chips. The samples with the RIN (RNA Integrity Number) closed to 10 were used for microarray. The SurePrint G3 Mouse GE8x60K Microarray Kit (Agilent Technologies, Santa Clara, CA) containing 62 976 oligonucleotide probes representing 39 430 genes was used. Quantile normalization was applied to sample data to correct for global intensity and dispersion. Then, a filtering at 75% was used to keep only genes expressed over the background noise, so 22 114 probes. A Significant Analysis of Microarray (SAM) 3 classes, with 10 000 permutations was applied with a False Discovery Rate (FDR) at 5% to look for mutant specific variation in gene expression in the dataset. Hierarchical clustering of the differentially expressed genes was obtained with TM4 Microarray Software Suite V4.9 (https://www.tm4.org) using average linkage clustering metrics and Pearson correlation for the distance. We identified enriched functional annotations for the clusters using DAVID Bioinformatics Resources 6.7 (https://david.ncifcrf.gov). A SAM 2 classes with a FDR at 5% was performed for Venn diagrams in TM4 and Cytoscape (http://www.cytoscape.org) was used to design the gene network with genes whose fold change is ≥ 2. The data discussed in this publication have been deposited in NCBI's Gene Expression Omnibus [63] and are accessible through GEO Series accession number GSE113251 (https://www.ncbi.nlm.nih.gov/geo/query/acc.cgi?acc=GSE113251).

### qPCR

On the same cDNA samples used for microarray experiments, we performed quantitative real-time PCR (qPCR) analyses of genes and housekeeping genes using SYBR Green PCR master mix (Applied Bio-systems, Life technologies, UK) on the Stratagene Mx3000P (Agilent Technologies).

### Statistical analysis

Data are expressed as means ± standard error of the mean (SEM). Significant differences between groups were determined using one-way or two-way analysis of variance (ANOVA) followed by Sidak post-hoc testing with Graphpad Prism software (Graphpad Prism 7.0, La Jolla, CA, USA). A p value <0.05 was considered statistically significant.

## Supporting information

S1 Fig Morphological, anatomical, and histological analysis of hypertrabeculated hearts in four-chamber views. (A) Long-axis cine images recorded by CMR at end-systole and end-diastole of 3 months-old control (CTL), *Nkx2-5*^*ΔTrbE10*^ (∆TrbE10) and *Nkx2-5*^*ΔTrbE14*^ (∆TrbE14) mutant mice. Arrows indicate excessive trabeculations. RV: right ventricle; IVS: interventricular septum; LV: left ventricle. (B) Anatomical structure of opened left ventricle of 12 months-old control, *Nkx2-5*^*ΔTrbE10*^ and *Nkx2-5*^*ΔTrbE14*^ mutant mice. The dotted lines delimit the free wall (LV) on the left and the interventricular septum (IVS) on the right. Stars indicate the papillary muscles. (C) Immunofluorescence with endomucin and RFP or endoglin and SMA antibodies to delineate the endocardium and capillaries from the arterial vasculature on sagittal sections of control, *Nkx2-5*^*ΔTrbE10*^ and *Nkx2-5*^*ΔTrbE14*^ adult hearts. On the right panels, high magnifications of the left ventricular lumen show the numerous endocardial islets in *Nkx2-5*^*ΔTrbE10*^ and *Nkx2-5*^*ΔTrbE14*^ mutants (arrows). (D) Immunofluorescence with WGA-cy3 to delineate fibrosis on sagittal sections of control, *Nkx2-5*^*ΔTrbE10*^ and *Nkx2-5*^*ΔTrbE14*^ adult hearts. Scale bar = 1mm.
(TIF)

S2 Fig Normal morphology of the central conduction system in Nkx2-5 mutants. Immunofluorescence with Contactin-2 (A-F) or Nkx2-5 (A’-F’) and WGA on serial sagittal sections at the level of the atrioventricular bundle (AVB) or atrioventricular node (AVN) from control (Ctrl), *Nkx2-5*^*ΔTrbE10*^ (∆TrbE10) and *Nkx2-5*^*ΔTrbE14*^ (∆TrbE14) adult hearts. Scale bar = 100μm (A-F) 50μm (A’-F’).
(TIF)

S3 Fig Subendocardial fibrosis in *Nkx2-5ΔTrbE10* and *Nkx2-5*^*ΔTrbE14*^ mutants. Trichrome masson coloration (A) or immunofluorescence with Vimentin and fibronectin antibodies (B-D) on transversal sections at the mid-ventricular level from control (Ctrl), *Nkx2-5*^*ΔTrbE10*^ and *Nkx2-5*^*ΔTrbE14*^ adult hearts. Scale bar = 100μm.
(TIF)

S4 Fig Graphs representing the longitudinal follow-up of the EF recorded by MRI in individual control, *Nkx2-5^ΔTrbE10^* and *Nkx2-5^ΔTrbE14^* mice. (TIF)

S5 Fig Quantification of right ventricular function in 3 and 9 month-old mice. Tricuspid annular plane systolic excursion (TAPSE) measurement by echocardiography shows right ventricular (RV) dysfunction in *Nkx2-5*^*ΔTrbE10*^ and *Nkx2-5*^*ΔTrbE14*^ mice at 9 month-old but not at 3-month-old of age (n = 6–7 per group) *, p<0.01 *vs* control mice.
(TIF)

S6 Fig Molecular markers associated with hypertrabeculated *Nkx2-5* mutant hearts and mouse models of pathological LVNC. Quantitative real-time PCR performed for a list of selected genes. The housekeeping gene used was RPL32.
(TIF)

S1 Movie Short-axis cine videos recorded by MRI of a control mouse. Compilation of three representative movies from MRI recordings in short axis of the same animal at the time points (3, 6 and 9 month-old) for a control mouse.
(MP4)

S2 Movie Short-axis cine videos recorded by MRI of a Nkx2-5^*ΔTrbE10*^ mouse. Compilation of three representative movies from MRI recordings in short axis of the same animal at the time points (3, 6 and 9 month-old) for a Nkx2-5^*ΔTrbE10*^ mouse.
(MP4)

S3 Movie Short-axis cine videos recorded by MRI of a Nkx2-5^*ΔTrbE14*^ mouse. Compilation of three representative movies from MRI recordings in short axis of the same animal at the time points (3, 6 and 9 month-old) for a *Nkx2-5*^*ΔTrbE14*^ mouse.
(MP4)

S4 Movie Short-axis cine videos recorded by MRI of a fluctuating Nkx2-5^*ΔTrbE10*^ mouse. Compilation of three representative movies from MRI recordings in short axis of the same animal at the time points (3, 6 and 9 month-old) for a fluctuating Nkx2-5^*ΔTrbE10*^ mouse.
(MP4)

S1 File Excel file with values extracted from the microarrays analysis. (XLSX)

S2 File Excel file with values from all quantifications. (XLSX)
