# Correction: Deletion of Nkx2-5 in trabecular myocardium reveals the developmental origins of pathological heterogeneity associated with ventricular non-compaction cardiomyopathy

**DOI:** 10.1371/journal.pgen.1007610

**Published:** 2018-08-15

**Authors:** Caroline Choquet, Thi Hong Minh Nguyen, Pierre Sicard, Emeline Buttigieg, Thi Thom Tran, Frank Kober, Isabelle Varlet, Rachel Sturny, Mauro W. Costa, Richard P. Harvey, Catherine Nguyen, Pascal Rihet, Sylvain Richard, Monique Bernard, Robert G. Kelly, Nathalie Lalevée, Lucile Miquerol

The following information is missing from the Funding section: This study was supported by GRRC (https://sfcardio.fr/grrc/sommaire) to CC.
