# Supplementary figures and images for: Deletion of Nkx2-5 in trabecular myocardium reveals the developmental origins of pathological heterogeneity associated with ventricular non-compaction cardiomyopathy
Source: PLoS Genet. 2018 Jul 6;14(7):e1007502. doi: 10.1371/journal.pgen.1007502 (PMC6051668; doi:10.1371/journal.pgen.1007502)

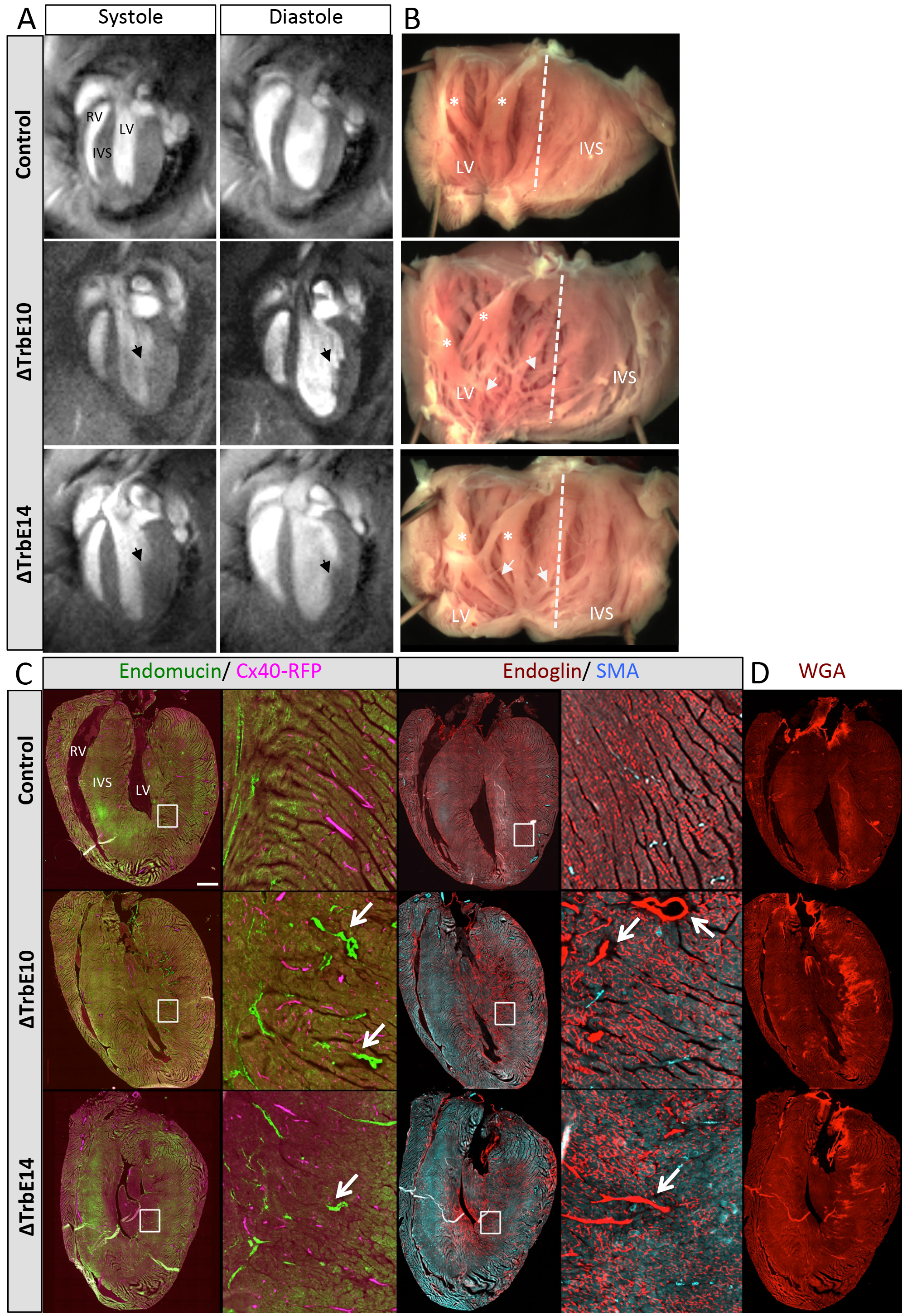

Supplement: S1 Fig — (A) Long-axis cine images recorded by CMR at end-systole and end-diastole of 3 months-old control (CTL), Nkx2-5ΔTrbE10 (∆TrbE10) and Nkx2-5ΔTrbE14 (∆TrbE14) mutant mice. Arrows indicate excessive trabeculations. RV: right ventricle; IVS: interventricular septum; LV: left ventricle. (B) Anatomical structure of opened left ventricle of 12 months-old control, Nkx2-5ΔTrbE10 and Nkx2-5ΔTrbE14 mutant mice. The dotted lines delimit the free wall (LV) on the left and the interventricular septum (IVS) on the right. Stars indicate the papillary muscles. (C) Immunofluorescence with endomucin and RFP or endoglin and SMA antibodies to delineate the endocardium and capillaries from the arterial vasculature on sagittal sections of control, Nkx2-5ΔTrbE10 and Nkx2-5ΔTrbE14 adult hearts. On the right panels, high magnifications of the left ventricular lumen show the numerous endocardial islets in Nkx2-5ΔTrbE10 and Nkx2-5ΔTrbE14 mutants (arrows). (D) Immunofluorescence with WGA-cy3 to delineate fibrosis on sagittal sections of control, Nkx2-5ΔTrbE10 and Nkx2-5ΔTrbE14 adult hearts. Scale bar = 1mm. (TIF) [file pgen.1007502.s001.tif]

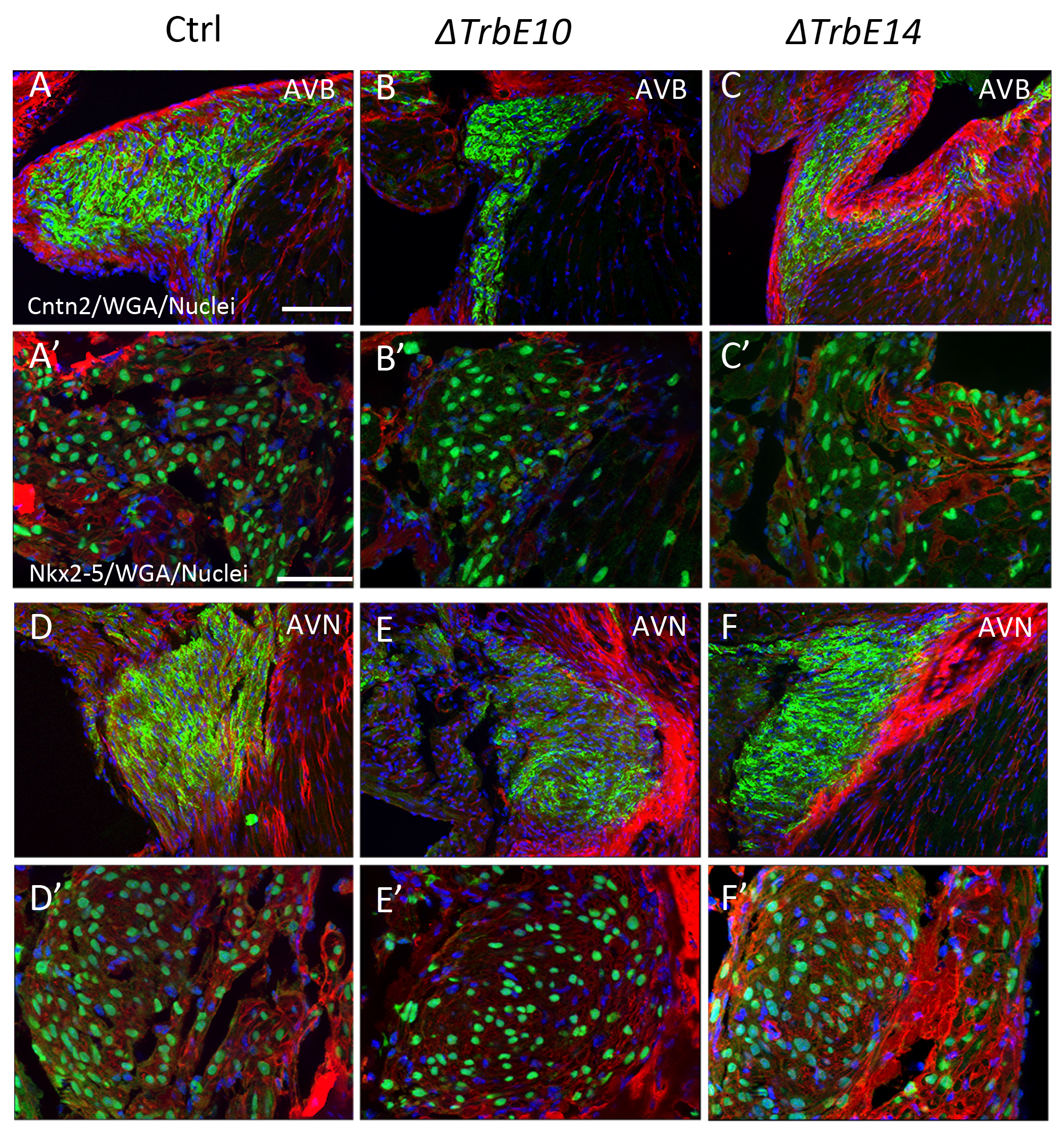

Supplement: S2 Fig — Immunofluorescence with Contactin-2 (A-F) or Nkx2-5 (A’-F’) and WGA on serial sagittal sections at the level of the atrioventricular bundle (AVB) or atrioventricular node (AVN) from control (Ctrl), Nkx2-5ΔTrbE10 (∆TrbE10) and Nkx2-5ΔTrbE14 (∆TrbE14) adult hearts. Scale bar = 100μm (A-F) 50μm (A’-F’). (TIF) [file pgen.1007502.s002.tif]

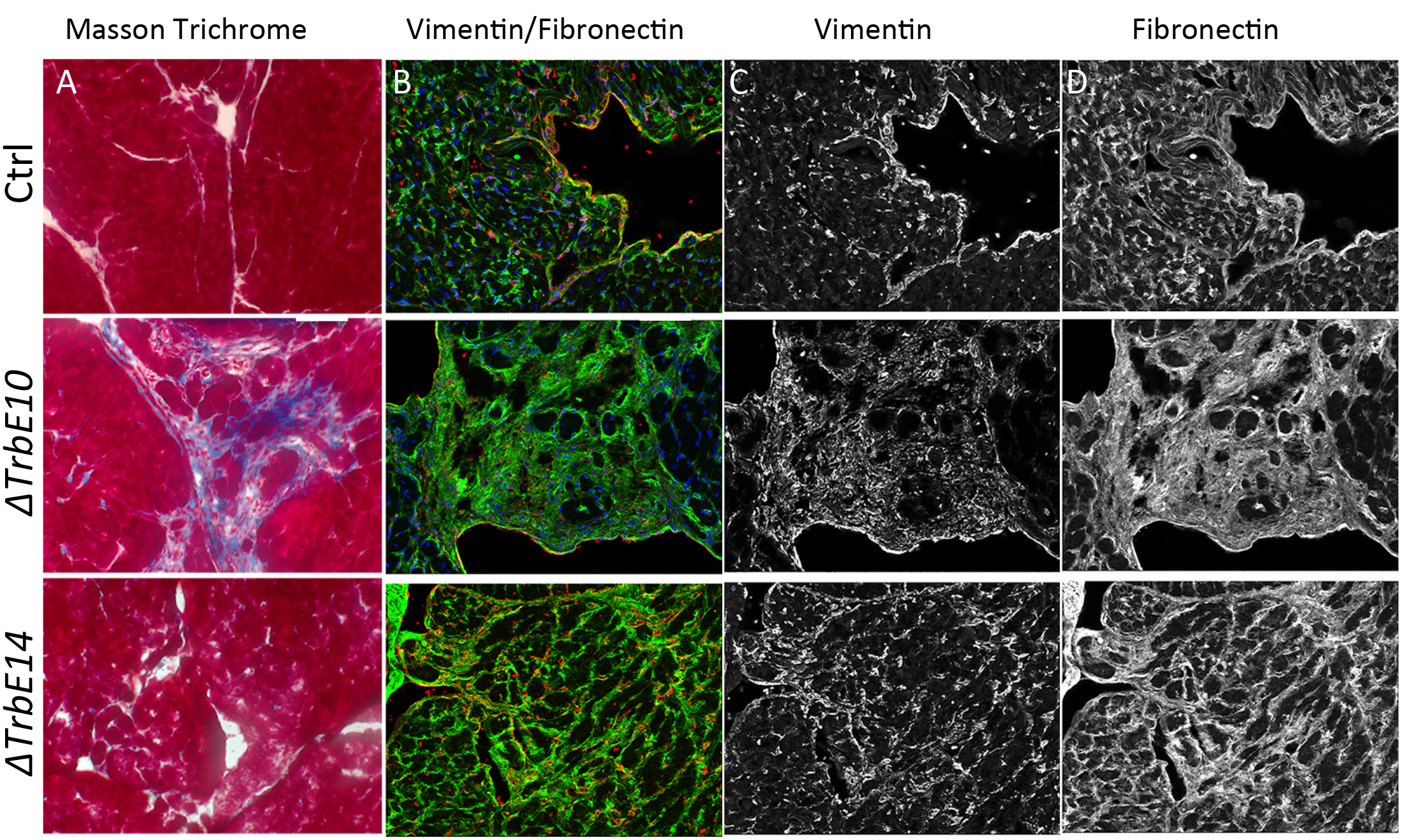

Supplement: S3 Fig — Trichrome masson coloration (A) or immunofluorescence with Vimentin and fibronectin antibodies (B-D) on transversal sections at the mid-ventricular level from control (Ctrl), Nkx2-5ΔTrbE10 and Nkx2-5ΔTrbE14 adult hearts. Scale bar = 100μm. (TIF) [file pgen.1007502.s003.tif]

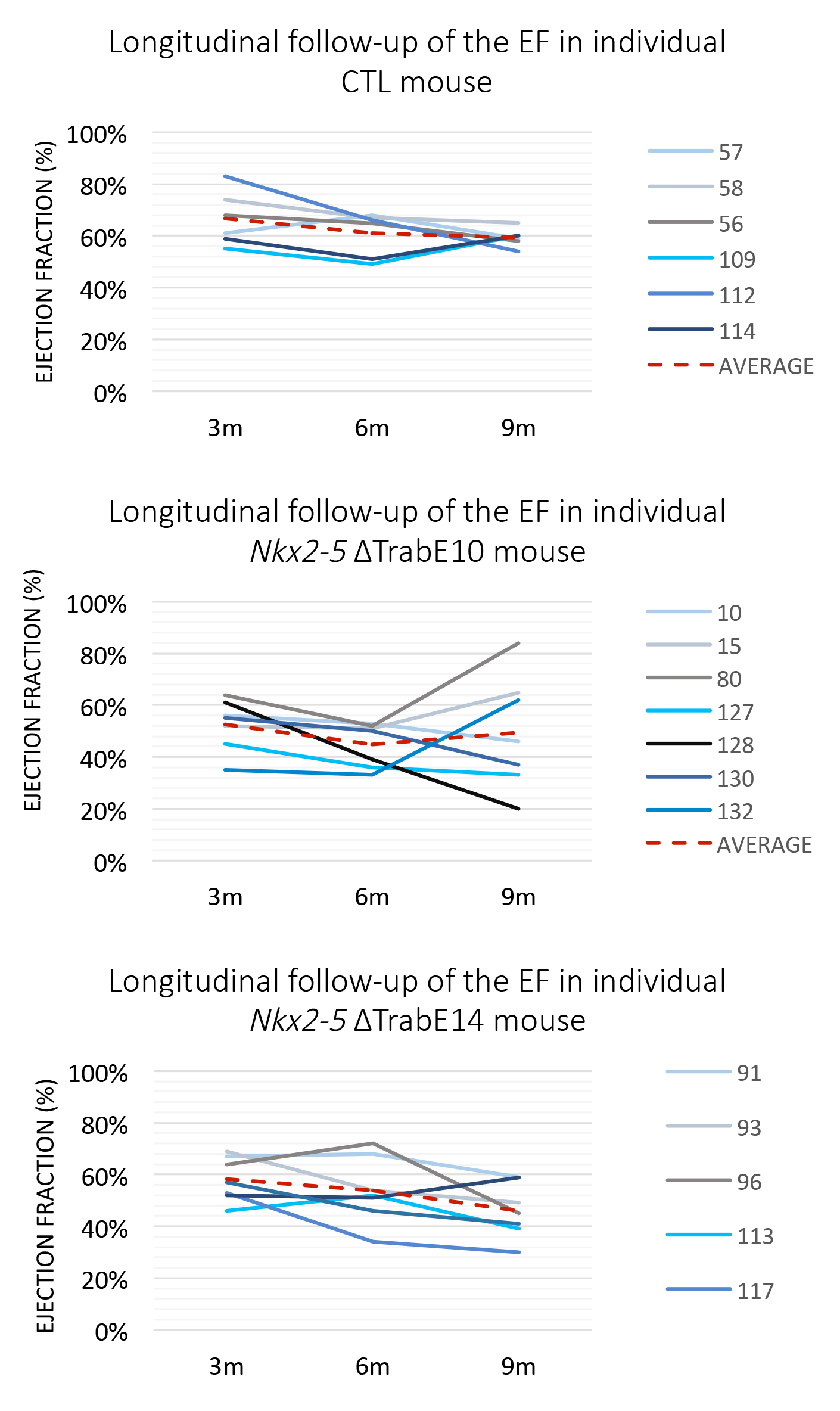

Supplement: S4 Fig — (TIF) [file pgen.1007502.s004.tif]

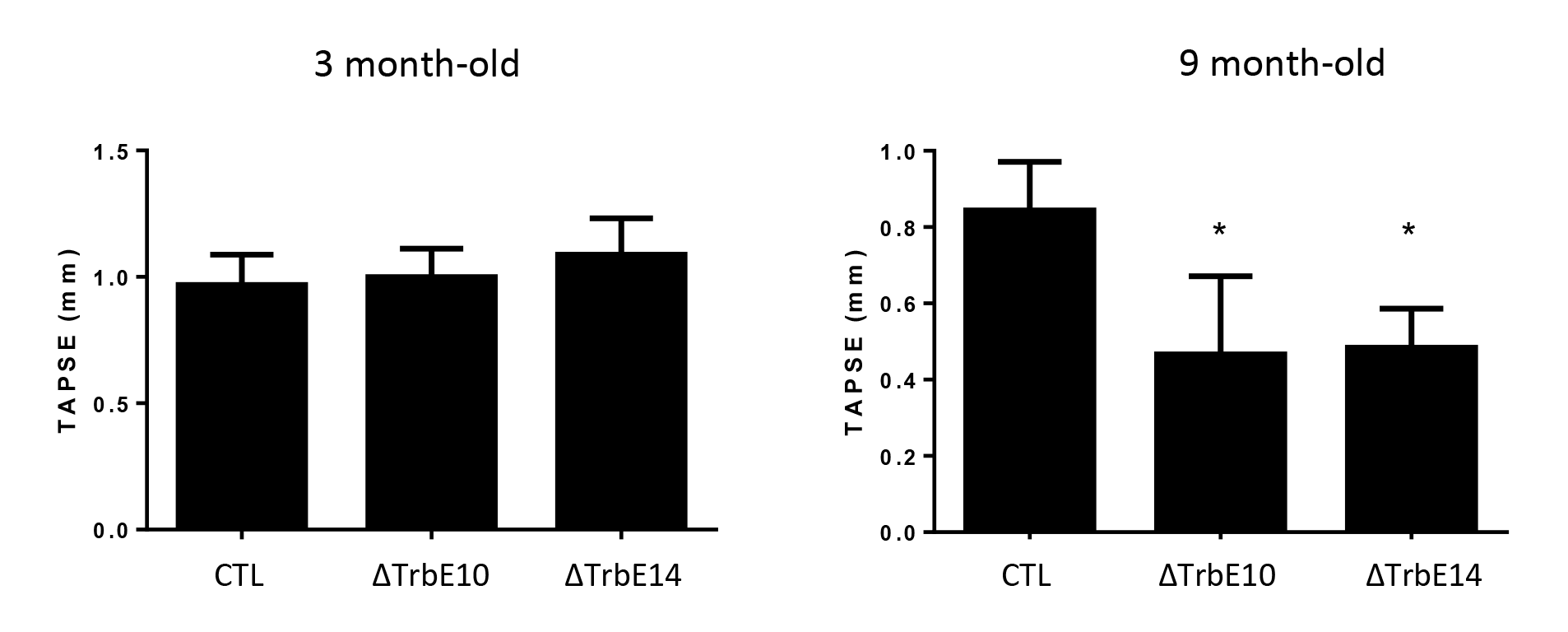

Supplement: S5 Fig — Tricuspid annular plane systolic excursion (TAPSE) measurement by echocardiography shows right ventricular (RV) dysfunction in Nkx2-5ΔTrbE10 and Nkx2-5ΔTrbE14 mice at 9 month-old but not at 3-month-old of age (n = 6–7 per group) *, p<0.01 vs control mice. (TIF) [file pgen.1007502.s005.tif]

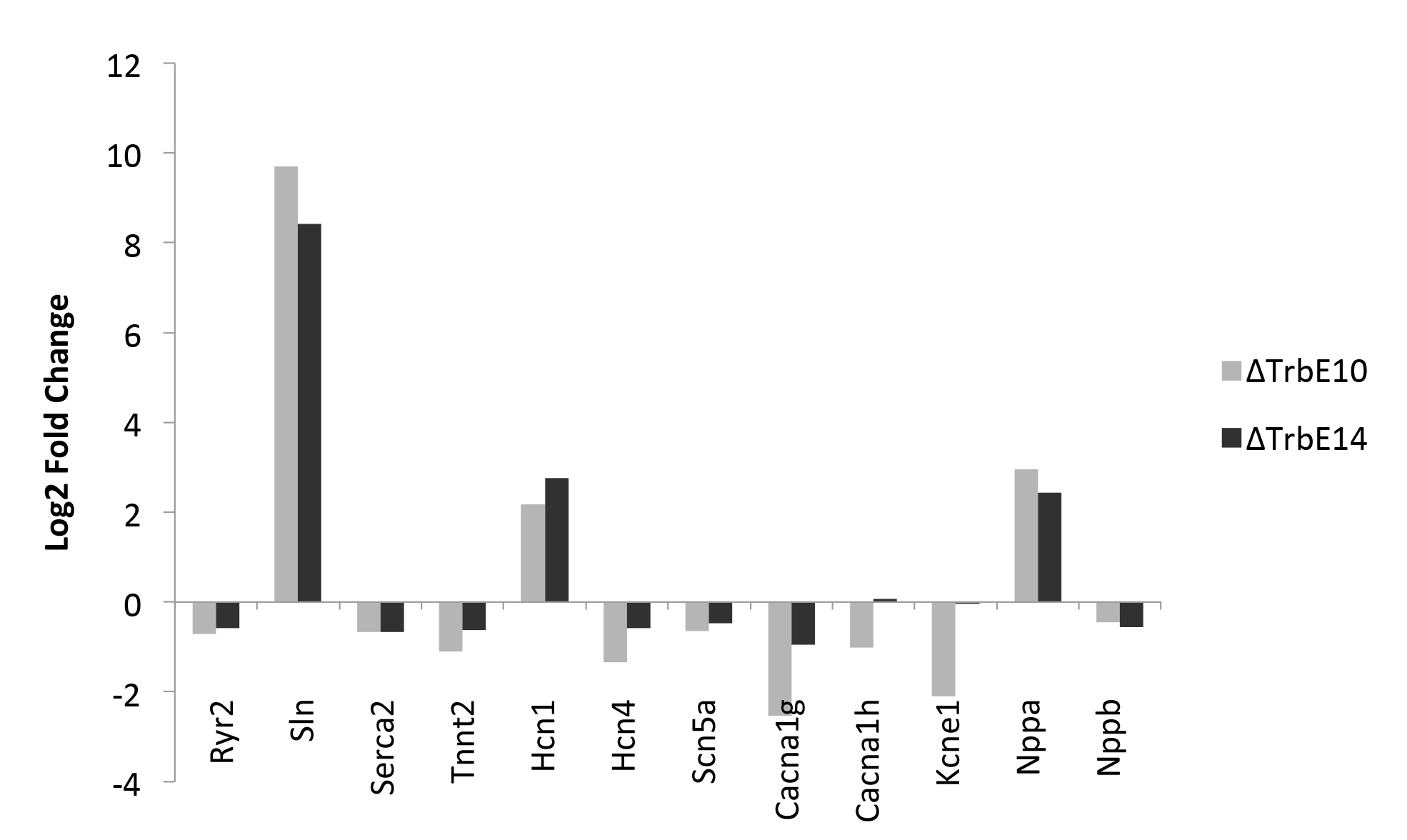

Supplement: S6 Fig — Quantitative real-time PCR performed for a list of selected genes. The housekeeping gene used was RPL32. (TIF) [file pgen.1007502.s006.tif]
